# Efficiency Enhancement in Testing Treatment Efficacy Across Multiple Populations Using Treatment Crossover Data

**DOI:** 10.1002/sim.70651

**Published:** 2026-07-15

**Authors:** Ryo Emoto, Kiyoaki Ishii, Toshinari Takamura, Shigeyuki Matsui

**Affiliations:** ^1^ Department of Biostatistics and Data Science, Graduate School of Medicine Kyoto University Kyoto Japan; ^2^ Department of Endocrinology and Metabolism, Graduate School of Medical Sciences Kanazawa University Kanazawa Japan; ^3^ Department of Musculoskeletal Disease National Center for Geriatrics and Gerontology Obu Japan; ^4^ Department of Interdisciplinary Statistical Mathematics The Institute of Statistical Mathematics Tokyo Japan

**Keywords:** crossover trials, personalized medicine, required sample size, statistical power, treatment effect heterogeneity

## Abstract

Recent advances in biotechnology and personalized medicine have driven the development of efficient clinical trial methodologies for assessing treatment efficacy across multiple populations defined by treatment effect modifiers. Within‐patient comparison of different treatments is a promising approach for improving study efficiency across multiple populations by eliminating between‐patient variability in treatment evaluation. This study provides a framework for evaluating treatment efficacy in multiple populations for 2×2 crossover trials and evaluates the efficacy gain in comparison with the standard parallel‐group analysis. Simulation experiments confirm that the crossover analysis consistently outperforms the parallel‐group analysis in statistical power, especially when carryover effects are small. An application to a clinical trial in Type 2 diabetes demonstrates the efficiency advantages of the crossover analysis. These numerical results emphasize the potential of the crossover analysis for enhancing the efficiency of clinical development of personalized medicine.

## 1 Introduction

Recent advances in biotechnology have led to a better understanding of diseases at the molecular level as well as the development of personalized medicine. Accordingly, the statistical methodologies for the design and analysis of clinical trials have evolved for the evaluation of treatment efficacy across multiple populations defined by baseline treatment effect modifiers, such as predictive markers. Specifically, in a randomized clinical trial with a predictive marker, the experimental treatment arm is compared with its control treatment arm in a marker‐defined subpopulation as well as in the overall population, with an appropriate false positive control for the multiple testing [1, 2, 3, 4].

A potential issue in such a parallel‐group comparison for multiple populations is the limited analytical efficiency, that is, a large number of patients are required to ensure sufficient precision in the statistical inference of treatment efficacy. By contrast, crossover clinical trials have traditionally been conducted as self‐controlled and efficient designs with much smaller sample sizes than parallel‐group trials to assess the main effects in short‐term responses of treatments with minimal carryover effects in the context of stable diseases [5, 6]. The efficiency gain is determined by comparing response outcomes under different treatments within patients, such that the nuisance factor of inter‐patient heterogeneity in response to the control treatment no longer affects treatment comparisons. The advantages of intra‐patient comparisons in terms of increasing efficiency also apply to the evaluation of treatment effect modification. The evaluation of treatment effect modification using crossover trial data has been considered, including double or replicate crossover trials [7, 8] as well as the integration of data from prior population‐level crossover studies with individual patient data [9].

Recently, Emoto et al. [10] proposed the use of specific treatment effect modifiers or predictive markers identified on the basis of biological or clinical hypotheses in a two‐sequence, two‐period (2×2) crossover trial. They provided a statistical framework to test and estimate the heterogeneity in treatment efficacy as a function of these predictive markers. Several transformations of the response variable were proposed to avoid modeling nuisance factors related to between‐patient variability and temporal transitions in the response variable for limited sample sizes in crossover trials. In contrast, this study considers a special case of the general model provided by Emoto et al. [10] for the most basic and common setting where a single binary predictive marker is used to evaluate treatment effects in the two populations, that is, a marker‐defined subpopulation and the overall population (the union of the marker‐defined and its complementary subpopulations). We then focus on the comparison in power and required sample size to test treatment effects in these populations between crossover and parallel‐group designs. Reducing the sample size or number of patients (rather than the number of repeated measurements per patient) is critically important, because crossover designs are often employed in clinical trials with small sample sizes.

The remainder of this paper is organized as follows. Section 2 introduces statistical models and methods for testing and estimating treatment efficacy in the overall and marker‐defined populations in both crossover and parallel‐group analyses. Section 3 presents numerical results on the statistical power and required sample size for evaluating the efficacy gain of using crossover analysis compared to the parallel‐group analysis. Section 4 presents numerical results using parameter estimates derived from actual clinical trial data for Type 2 diabetes, highlighting the practical implications of the proposed methods. Finally, Section 5 discusses the findings of the study and concludes the paper.

## 2 Statistical Models and Inference of Treatment Efficacy

We compare an experimental treatment with its control treatment in a 2×2 crossover trial involving two populations, that is, an overall population and a subpopulation defined by a baseline predictive marker as a treatment effect modifier. Here, the marker‐defined subpopulation, typically referred to as a “marker‐positive” subpopulation, is anticipated to exhibit a significant benefit from the experimental treatment based on biological or clinical hypotheses on this marker. The objective of the primary analysis of this clinical trial is to demonstrate treatment efficacy in either the overall population or the marker‐defined subpopulation. Accordingly, an appropriate false positive control will be established for the analysis of these two populations.

### 2.1 Statistical Models

In the 2×2 crossover trial, the treatment sequence $\left(A_{i1},A_{i2}\right)$ randomly assigned to patient i (i=1,…,n) is either (1,−1) or (−1,1), representing either “experimental treatment followed by control treatment” or “control treatment followed by experimental treatment,” respectively. In general, a washout period is introduced between successive treatment periods to minimize the carryover effects of the first treatment during the second treatment period. Let $Y_{ij}$ be a continuous response variable that represents a response after the assigned treatment $A_{ij}$ during treatment period j (j=1,2). For each treatment period, the response variable is typically specified as the change in a variable representing disease status before and after treatment (e.g., the change in the HbA1c level before and after drug treatment in a diabetes trial, see Section 4). We assume that $Y_{ij}$ is defined such that higher values indicate better outcomes for patients. To define the subpopulation membership, reflecting treatment effect modification across patients, we introduce a binary baseline marker $x_{i}$ ∈{0,1} for patient i, such that the marker‐defined subpopulation corresponds to the group of patients with $x_{i}=1$ and the complementary subpopulation to the group with $x_{i}=0$. Here, we consider evaluating treatment effects in the marker‐defined subpopulation ($x_{i}=1$) and in the overall population ($x_{i}\in \{0,1\}$, the union of the marker‐defined and its complementary subpopulations), as seen in many clinical trials with a binary marker. By introducing multiple marker covariates, we can consider treatment effects in multiple populations, such as those defined by two or more cut‐offs for a single continuous marker and those based on multiple marker variables. However, in this paper we focus on the comparison of crossover and parallel‐group designs in the case with a single marker‐defined subpopulation and the overall population, since it is the most basic and common setting in marker‐based clinical trials.

The fundamental assumption for using a crossover trial is that patients will be in the same state at the start of the second period (j=2) as they were in at the start of the first period (j=1) [5]. We make this assumption for the marker variable $x_{i}$, so that $x_{i}$ can serve as a predictive marker for the response variable in the second period $Y_{i2}$, as well as for the response variable in the first period $Y_{i1}$. Such a marker may capture the underlying stable disease state of individual patients. Specifically, for patient i (i=1,⋯,n) and treatment period j (j=1,2), we assume the following model:

(1) $$\begin{array}{l} Y_{ij}=m_{i}+\frac{A_{ij}}{2}(\theta_{0}+\theta_{1}x_{i})+\phi_{ij}\left\{t_{i}+\frac{A_{ij}}{2}(\gamma_{0}+\gamma_{1}x_{i})\right\}+e_{ij}, \end{array}$$

where $\phi_{ij}=0$ for treatment period j=1 and $\phi_{ij}=1$ for treatment period j=2. This model includes three random variation terms, namely $m_{i}$, $t_{i}$, and $e_{ij}$, where $m_{i}$ represents a patient‐specific random effect on the response variable with $E[m_{i}]=\mu_{m}$ and $\mathrm{Var}(m_{i})=\sigma_{m}^{2}$, $t_{i}$ represents a random effect on the response variable that appears in the second period, with $E[t_{i}]=\mu_{t}$ and $\mathrm{Var}(t_{i})=\sigma_{t}^{2}$, and $e_{ij}$ is a random error with $E[e_{ij}]=0$, $\mathrm{Var}(e_{ij})=\sigma_{e}^{2}$, and $\text{corr}(e_{i1},e_{i2})=0$.

The expected patient‐level response $E[Y_{ij}|m_{i},t_{i},A_{ij}]$ is summarized in Table 1. The first component in (1), $m_{i}+\frac{A_{ij}}{2}(\theta_{0}+\theta_{1}x_{i})$, represents the expected response in the first treatment period. Here, the component $\theta_{0}+\theta_{1}x_{i}$ serves as the kernel in evaluating treatment efficacy in the overall and marker‐defined populations. To see this, the contrast of the two expected responses under different treatments in the first treatment period, $E[Y_{i1}|m_{i},t_{i},A_{i1}=1]-E[Y_{i1}|m_{i},t_{i},A_{i1}=-1]$, reduces to $\theta_{0}+\theta_{1}x_{i}$. For the marker‐defined subpopulation with $x_{i}=1$, the treatment effect is expressed as $\theta_{0}+\theta_{1}$, whereas for its complementary subpopulation with $x_{i}=0$, the treatment effect is expressed as $\theta_{0}$. The treatment effect in the overall population is a weighted average of these two effects, $p(\theta_{0}+\theta_{1})+(1-p)\theta_{0}=\theta_{0}+p\theta_{1}$, where p is the prevalence of the marker‐defined subpopulation in the clinical trial. We define $\delta_{0},\delta_{1}$, and $\delta_{2}$ as the average of the treatment effects in the complementary subpopulation, marker‐defined subpopulation, and overall population, respectively:

$$\begin{array}{l} \delta_{0}=\theta_{0},\delta_{1}=\theta_{0}+\theta_{1},\;\text{and}\;\delta_{2}=\theta_{0}+p\theta_{1}. \end{array}$$

**TABLE 1:** Expected patient‐level response in (1).

Sequence	First period	Second period
1 $\left(A_{i1},A_{i2})=(1,-1\right)$	$m_{i}+(\theta_{0}+\theta_{1}x_{i})/2$	$m_{i}-(\theta_{0}+\theta_{1}x_{i})/2+t_{i}-(\gamma_{0}+\gamma_{1}x_{i})/2$
2 $\left(A_{i1},A_{i2})=(-1,1\right)$	$m_{i}-(\theta_{0}+\theta_{1}x_{i})/2$	$m_{i}+(\theta_{0}+\theta_{1}x_{i})/2+t_{i}+(\gamma_{0}+\gamma_{1}x_{i})/2$

The second component in (1), $t_{i}+\frac{A_{ij}}{2}(\gamma_{0}+\gamma_{1}x_{i})$, is added to modify the first component by treatment crossover, where $\gamma_{0}$ and $\gamma_{1}$ represent carryover effects pertaining to the main effect of treatment $\theta_{0}$ and the treatment effect modification by the marker (or treatment‐marker interaction) $\theta_{1}$, respectively.

Another way to derive the kernel $\theta_{0}+\theta_{1}x_{i}$ is to incorporate crossover data in the second treatment period. We consider the contrasts of the two response expectations between two treatment periods (with different treatments) within patients. For example, with the first treatment sequence $\left(A_{i1},A_{i2})=(1,-1),E[Y_{i1}|m_{i},t_{i},A_{i1},A_{i2}]-E[Y_{i2}|m_{i},t_{i},A_{i1},A_{i2}\right]$ reduces to $(\theta_{0}+\theta_{1}x_{i})-t_{i}+(\gamma_{0}+\gamma_{1}x_{i})/2$. In the next subsection, we consider transformations of the response variable to evaluate the kernel $\theta_{0}+\theta_{1}x_{i}$, incorporating the time effect $t_{i}$ and carryover component $\gamma_{0}+\gamma_{1}x_{i}$.

The notation and statistical model above are general and different from the traditional ones used to analyze the main treatment effects in the 2×2 crossover design without the evaluation of treatment effect modification. For those who are familiar with the traditional notation and statistical models, see Section S3.6 in the Supporting Information that maps the fixed and random effects used in this section to those in the standard 2×2 crossover cell means model. One reason for using the general model is that it allows us to explicitly model treatment effect modification based on the marker $x_{i}$ via fixed effects, while grouping the other nuisance factors as random components. Another reason is that the general model allows a clear comparison between crossover and parallel‐group designs. In particular, the specification of non‐zero means for the random effects ($m_{i},t_{i}$) will highlight the difference in the impact of the non‐zero means on the operating characteristics between these two designs, as shown in Section 3.

### 2.2 Crossover Analysis to Evaluate Treatment Efficacy

For the response variable $\left(Y_{i1},Y_{i2}\right)$ of patient i, we consider the following transformation of within‐patient comparison: $R_{i}=G_{i}(Y_{i1}-Y_{i2})$, where $G_{i}=-1$ if the treatment sequence (−1,1) is assigned to the patient and $G_{i}=1$ otherwise [10]. Following Emoto et al. [10], this transformation is designed to remove the patient‐specific random effect $m_{i}$ and simplify the model for the treatment effect kernel $\psi (x_{i})$, assuming negligible carryover effects. Based on (1), we have the following model for $R_{i}$:

(2) $$\begin{array}{ll} R_{i} & =\psi (x_{i})+\varepsilon_{i}, \end{array}$$

where

$$\begin{array}{ll} \psi (x_{i}) & =\psi_{0}+\psi_{1}x_{i}\\ \psi_{k} & =\theta_{k}+\frac{1}{2}\gamma_{k}\;(k=0,1), \end{array}$$

and the error term is $\varepsilon_{i}=-G_{i}\left(t_{i}-e_{i1}+e_{i2}\right)$. We regard the treatment sequence indicator $G_{i}$ as a random variable rather than a fixed one. Assume equal allocation to treatment sequences, that is, $\mathrm{Pr}(G_{i}=-1)=\mathrm{Pr}(G_{i}=1)=1/2$, such that $E[G_{i}]=0$. Note that the transformation of the response variable $R_{i}$ used in the crossover analysis can be extended to accommodate unequal allocation ratios (see Section S3.5 in the Supporting Information). However, we focus on equal allocation in this study as it represents the most general and standard setting of actual clinical trial designs. Since the assignment of treatment sequence $G_{i}$ is independent of the random time effect in the second period $t_{i}$ and the random errors $e_{i1}$ and $e_{i2}$, the error term $\varepsilon_{i}$ has a distribution with $E[\varepsilon_{i}]=0$ and $\mathrm{Var}(\varepsilon_{i})=\mu_{t}^{2}+\sigma_{t}^{2}+2\sigma_{e}^{2}$ (see Section S3.1 in the Supporting Information).

When the carryover effects $\gamma_{0}$ and $\gamma_{1}$ are considered small based on arguments on the presumed mechanisms of the disease and treatment action, adequacy of the length of the washout period, and so on, we may consider an estimate for $\psi (x_{i})$ with $\gamma_{0}=\gamma_{1}=0$ as a reasonable estimate for the kernel $\theta_{0}+\theta_{1}x_{i}$. Let ${\hat{\psi }}_{0}$ and ${\hat{\psi }}_{1}$ be estimators of $\psi_{0}$ and $\psi_{1}$ when (2) is fitted to the crossover data using a least‐squares method. Then, we obtain the following estimators for the treatment effect parameters: $\delta_{1}$ in the marker‐defined subpopulation and $\delta_{2}$ in the overall population (see Section S3.2 in the Supporting Information).

(3) $$\begin{array}{cc} {\hat{\delta }}_{1}^{\text{CO}} & ={\hat{\psi }}_{0}+{\hat{\psi }}_{1}=\frac{1}{np}\overset{n}{\underset{i=1}{\sum }}x_{i}R_{i},\\ {\hat{\delta }}_{2}^{\text{CO}} & ={\hat{\psi }}_{0}+p{\hat{\psi }}_{1}=\frac{1}{n}\overset{n}{\underset{i=1}{\sum }}R_{i}. \end{array}$$

Notably, under the standard assumption of equal allocation, these estimators may reduce to one‐half of the difference in the average of the within‐patient change (i.e., period difference $Y_{i1}-Y_{i2}$) between the two arms of the treatment sequence in the respective populations. This indicates that our modeling framework provides treatment effect estimates corresponding to those obtained from the standard two‐sample t‐test approach commonly used in crossover trials. The variances of these estimators are given by

(4) $$\begin{array}{cc} \mathrm{Var}({\hat{\delta }}_{1}^{\text{CO}}) & =\mathrm{Var}(\varepsilon_{i})/np,\\ \mathrm{Var}({\hat{\delta }}_{2}^{\text{CO}}) & =\mathrm{Var}(\varepsilon_{i})/n. \end{array}$$

We can estimate these variances on the basis of an estimated variance of $\varepsilon_{i}$ obtained in the least‐squares fitting of the model of $R_{i}$ to the data.

When the assumption that carryover effects $\gamma_{0}$ and $\gamma_{1}$ are negligible is questionable, following Emoto et al. [10], we can estimate them on the basis of another transformation of the response variable, $S_{i}=-2G_{i}(Y_{i1}+Y_{i2})$. Under (1), $S_{i}$ can be modeled as $S_{i}=\gamma_{0}+\gamma_{1}x_{i}+\varepsilon_{i}^{\prime }$, where $\varepsilon_{i}^{\prime }=-2G_{i}(2m_{i}+t_{i}+e_{i1}+e_{i2})$ can be regarded as a zero‐mean random error under equal allocation of the treatment sequence, thereby avoiding the modeling of $m_{i}$ and $t_{i}$. With estimators ${\hat{\gamma }}_{0}$ and ${\hat{\gamma }}_{1}$ obtained by a least‐squares fitting of the regression model of $S_{i}$ to the data, we have another estimator of $\theta_{k}$, ${\hat{\theta }}_{k}^{*}={\hat{\psi }}_{k}-{\hat{\gamma }}_{k}/2$ (k=0,1). Here, it is interesting to note that this estimator based on the least‐squares estimation will reduce to an estimator obtained by the parallel‐group analysis using data from the first treatment period that is given in Section 2.3 (see Section S3.3 in the Supporting Information). Therefore, we compare the efficiency of the estimators under the assumption of negligible carryover effects with its counterpart in the parallel‐group analysis in Section 3.

As the basic assumption underlying the adoption of the crossover trial is that carryover effects are small, the estimators given in (3) will serve as the primary analysis for evaluating treatment efficacy in the marker‐defined and overall populations. Meanwhile, the estimators $\hat{\gamma_{0}}$ and $\hat{\gamma_{1}}$ can be used to check the assumption of no carryover effects, that is, $\gamma_{0}=0$ and $\gamma_{1}=0$. If the carryover effects are significant, one would choose the estimators incorporating the carryover effects or those from the parallel‐group analysis using data from the first treatment period alone.

### 2.3 Parallel‐Group Analysis for Treatment Efficacy in Multiple Populations

In the parallel‐group analysis in the first treatment period, we have the reduced model $Y_{i1}=m_{i}+\frac{1}{2}A_{i1}(\theta_{0}+\theta_{1}x_{i})+e_{i1}$. A simple approach for estimating the treatment effect parameters $\theta_{0}$ and $\theta_{1}$ without modeling the nuisance factors $m_{i}$ is to use the transformation $Z_{i}=2Y_{i1}A_{i1}$ [10, 11]. Under the standard equal allocation of treatment, $\mathrm{Pr}(A_{i1}=1)=\mathrm{Pr}(A_{i1}=-1)=1/2$, such that $E[A_{i1}]=0$, we have the following model:

(5) $$\begin{array}{ll} Z_{i} & =\theta_{0}+\theta_{1}x_{i}+\varepsilon_{i}^{\prime }, \end{array}$$

where $\varepsilon_{i}^{\prime }=2A_{i}(m_{i}+e_{i1})$ represents a random error with $E[\varepsilon_{i}^{\prime }]=0$ and $\mathrm{Var}(\varepsilon_{i}^{\prime })=4(\mu_{m}^{2}+\sigma_{m}^{2}+\sigma_{e}^{2})$ under independence of treatment assignment $A_{i}$ from $m_{i}$ and $e_{i1}$ (see Section S3.1 in the Supporting Information). In contrast to the crossover error term $\varepsilon_{i}$ in (2), the parallel‐group error term $\varepsilon_{i}^{\prime }$ includes the patient‐specific effect $m_{i}$, leading to a larger variance when between‐patient variability is substantial. The parameters $\theta_{0}$ and $\theta_{1}$ are estimated by a least‐squares fitting of the regression model (5) to the first‐period data. Using these estimators ${\hat{\theta }}_{0}$ and ${\hat{\theta }}_{1}$, we then derive the parallel‐group analysis estimators of treatment effects in the marker‐defined and overall populations (see Section S3.2 in the Supporting Information):

(6) $$\begin{array}{cc} {\hat{\delta }}_{1}^{\text{PG}} & ={\hat{\theta }}_{0}+{\hat{\theta }}_{1}=\frac{1}{np}\overset{n}{\underset{i=1}{\sum }}x_{i}Z_{i},\\ {\hat{\delta }}_{2}^{\text{PG}} & ={\hat{\theta }}_{0}+p{\hat{\theta }}_{1}=\frac{1}{n}\overset{n}{\underset{i=1}{\sum }}Z_{i}. \end{array}$$

The variances of these estimators are given by

(7) $$\begin{array}{cc} \mathrm{Var}({\hat{\delta }}_{1}^{\text{PG}}) & =\mathrm{Var}(\varepsilon_{i}^{\prime })/np,\\ \mathrm{Var}({\hat{\delta }}_{2}^{\text{PG}}) & =\mathrm{Var}(\varepsilon_{i}^{\prime })/n. \end{array}$$

We can estimate these variances on the basis of an estimated variance of $\varepsilon_{i}^{\prime }$ in the least‐squares fitting of the model of $Z_{i}$ to the data in the first treatment period.

### 2.4 Power and Sample Size Calculations

The null and alternative hypotheses in one‐sided testing of treatment effects in the marker‐defined and overall populations are expressed as follows:

(8) $$\begin{array}{ll} H_{1,0}:\delta_{1}\leq 0\; & \text{v.s.}\;H_{1,1}:\delta_{1}>0, \end{array}$$

(9) $$\begin{array}{ll} H_{2,0}:\delta_{2}\leq 0\; & \text{v.s.}\;H_{2,1}:\delta_{2}>0. \end{array}$$

Although many multiple testing procedures have been proposed for testing these two null hypotheses with both strong and weak controls of the alpha error rate [3, 4], the ultimate goal of marker‐based clinical trials will be the detection of treatment effects in either marker‐defined subpopulation or overall population. In other words, the probability of “success” in demonstrating treatment efficacy in any population will be mainly considered the statistical power to be ensured in marker‐based clinical trials. Let $T_{1}^{\text{CO}}$ and $T_{2}^{\text{CO}}$ be the test statistics used in the crossover analysis assuming negligible carryover effects to test the null treatment effects in the marker‐defined subpopulation and the overall population, respectively, such that $T_{h}^{\text{CO}}={\hat{\delta }}_{h}^{\text{CO}}/\sqrt{\mathrm{Var}({\hat{\delta }}_{h}^{\text{CO}})}$ (h=1,2). Similarly, let $T_{1}^{\text{PG}}$ and $T_{2}^{\text{PG}}$ denote the test statistics used in the parallel‐group analysis to test the null treatment effects in the marker‐defined subpopulation and the overall population, respectively, such that $T_{h}^{\text{PG}}={\hat{\delta }}_{h}^{\text{PG}}/\sqrt{\mathrm{Var}({\hat{\delta }}_{h}^{\text{PG}})}$ (h=1,2).

In statistical power and sample size calculations, we assume that these statistics follow the standard normal distribution. Here, the covariance (or correlation coefficient) between $T_{1}^{\text{CO}}$ and $T_{2}^{\text{CO}}$ reduces to $\sqrt{p}$ (see Section S3.4 in the Supporting Information). Thus, we assume the bivariate normal distribution for $T_{1}^{\text{CO}}$ and $T_{2}^{\text{CO}}$ with mean and variance as follows:

(10) $$\begin{array}{ll} & \mu^{\text{CO}}={\left(\frac{\delta_{1}+(\gamma_{0}+\gamma_{1})/2}{\sqrt{V^{\text{CO}}/(np)}},\frac{\delta_{2}+(\gamma_{0}+p\gamma_{1})/2}{\sqrt{V^{\text{CO}}/n}}\right)}^{T},\\ & \Sigma^{\text{CO}}=\left(\begin{array}{cc} 1 & \sqrt{p}\\ \sqrt{p} & 1 \end{array}\right), \end{array}$$

where $V^{\text{CO}}=\mathrm{Var}(R_{i})=\mathrm{Var}(\varepsilon_{i})=\mu_{t}^{2}+\sigma_{t}^{2}+2\sigma_{e}^{2}$.

To control the family‐wise error rate at level α for the testing of treatment effects for $H_{1,0}$ and $H_{2,0}$, let C(α) denote the rejection region corresponding to a chosen multiple testing procedure. Under alternative treatment effects $\delta_{1}=d_{1}(>0)$ and $\delta_{2}=d_{2}(>0)$, the probability of rejecting at least one of the null hypotheses, $H_{1,0}$ or $H_{2,0}$, is expressed using the joint probability density function $f(t_{1}^{\text{CO}},t_{2}^{\text{CO}};\delta_{1},\delta_{2})$ as:

(11) $$\begin{array}{ll} \Psi (d_{1},d_{2};C(\alpha )) & =\iint_{\left(t_{1}^{\text{CO}},t_{2}^{\text{CO}})\in C(\alpha \right)}f(t_{1}^{\text{CO}},t_{2}^{\text{CO}};d_{1},d_{2})\;dt_{1}^{\text{CO}}\;dt_{2}^{\text{CO}}. \end{array}$$

The geometry of C(α) depends on the testing strategy. For instance, in the Bonferroni method, the region is the union of individual rejection areas: $C(\alpha )=\{(t_{1},t_{2}):t_{1}>z_{\alpha ⁄2}\;\text{or}\;t_{2}>z_{\alpha ⁄2}\}$. On the other hand, in a hierarchical testing procedure, for example, for testing treatment effects in the overall population only if the effect in the marker‐defined subpopulation is significant, the region for trial success simplifies to $C(\alpha )=\{(t_{1},t_{2}):t_{1}>z_{\alpha }\}$, because only the first test in the marker‐defined subpopulation is related to the statistical power defined above. In the numerical evaluation (Section 3), we adopt the Bonferroni method to compare the efficiency of the designs.

Similarly, for $T_{h}^{\text{PG}}$ (h=1,2), we assume the bivariate normal distribution with mean and variance as follows:

(12) $$\begin{array}{ll} \mu^{\text{PG}} & ={\left(\frac{\delta_{1}}{\sqrt{V^{\text{PG}}/(np)}},\frac{\delta_{2}}{\sqrt{V^{\text{PG}}/n}}\right)}^{T},\Sigma^{\text{PG}}=\left(\begin{array}{cc} 1 & \sqrt{p}\\ \sqrt{p} & 1 \end{array}\right), \end{array}$$

where $V^{\text{PG}}=\mathrm{Var}(Z_{i})=\mathrm{Var}(\varepsilon_{i}^{\prime })=4(\mu_{m}^{2}+\sigma_{m}^{2}+\sigma_{e}^{2})$. Again, the covariance between $T_{1}^{\text{PG}}$ and $T_{2}^{\text{PG}}$ is $\sqrt{p}$ (see Section S3.4 in the Supporting Information). The power of the multiple testing using $T_{h}^{\text{PG}}$ (h=1,2) can be expressed similarly to (11).

The required sample size is determined as a minimum value of n that achieves a desirable power level for given values of $p,d_{1},d_{2}$, and rejection region C(α).

## 3 Efficiency Comparison

In this section, we compare the statistical power and required sample size for the inference of treatment effects in the marker‐defined and overall populations between the crossover and parallel‐group analyses. As for the treatment effect parameters in multiple populations, we consider standardized effect sizes $\Delta_{0}=\delta_{0}/\sigma_{e}$ in the complementary subpopulation and $\Delta_{1}=\delta_{1}/\sigma_{e}$ in the marker‐defined subpopulation. We also consider standardized mean and variance parameter of random effects in $m_{i}$ and $t_{i}$, such that $\xi_{m}=\mu_{m}/\sigma_{m},\rho_{m}=\sigma_{m}^{2}/\sigma_{e}^{2},\xi_{t}=\mu_{t}/\sigma_{t},\rho_{t}=\sigma_{t}^{2}/\sigma_{e}^{2}$. For the carryover effects, we introduce κ as a common ratio of carryover effects (that appear in the second treatment period) for the main treatment effects and treatment‐marker interaction in the first treatment period, such that $\kappa =-\gamma_{k}/\theta_{k}$ (k=0,1), and we have the equation $\frac{A_{i2}}{2}(\gamma_{0}+\gamma_{1}x_{i})=\kappa \frac{A_{i1}}{2}(\theta_{0}+\theta_{1}x_{i})$ (note: $A_{i1}=-A_{i2}$). Using these parameters, the means of the test statistics, $T_{h}^{\text{CO}}$ and $T_{h}^{\text{PG}}$ (h=1,2) in (10) and (12), are expressed as follows:

$$\begin{array}{ll} \mu^{\text{CO}} & =\frac{1-\kappa /2}{\sqrt{\xi_{t}^{2}\rho_{t}+\rho_{t}+2}}{\left(\sqrt{np}\Delta_{1},\sqrt{n}\{(1-p)\Delta_{0}+p\Delta_{1}\}\right)}^{T},\\ \mu^{\text{PG}} & =\frac{1}{\sqrt{4(\xi_{m}^{2}\rho_{m}+\rho_{m}+1)}}{\left(\sqrt{np}\Delta_{1},\sqrt{n}\{(1-p)\Delta_{0}+p\Delta_{1}\}\right)}^{T}. \end{array}$$

The first formula indicates that a larger value of κ (large carryover effects) will produce small power values in the crossover analysis. We note that the mean of $T_{h}^{\text{CO}}$ depends on $\xi_{t}$ and $\rho_{t}$ of the time effect $t_{i}$, while that of $T_{h}^{\text{PG}}$ depends on $\xi_{m}$ and $\rho_{m}$ of the random effect $m_{i}$.

With respect to the profile of the treatment effects across populations, we consider the following three scenarios based on treatment effects within the marker‐defined population and its complementary subpopulation [12]:

1. Constant effects: $\Delta_{1}=\Delta_{0}>0$, indicating that the treatment effect is uniform across subpopulations.
2. Quantitative interaction: $\Delta_{1}>\Delta_{0}>0$, reflecting a stronger treatment effect in the marker‐positive subpopulation.
3. Qualitative interaction: $\Delta_{1}>0,\Delta_{0}=0$, implying that the treatment effect is only observed in the marker‐positive subpopulation.

These scenarios suppose clinical trials, including those to evaluate the efficacy of new treatments for placebo controls or treatments added to standard treatments for standard treatments alone. We also investigated scenarios of qualitative interaction with reversed effects ($\Delta_{1}>0$ and $\Delta_{0}<0$) supposing clinical trials to compare competitive treatments: However, similar trends were observed for relative efficiency gain by the crossover analysis compared to the parallel‐group analysis (see Section S1 in the Supporting Information). For these non‐null treatment effect scenarios for the two marker subpopulations, it is reasonable to assume that the null treatment scenario represents no treatment effects in both subpopulations, that is, $\Delta_{0}=0$ and $\Delta_{1}=0$. For this scenario, it is reasonable to assume that carryover effects of treatment in these populations, $\gamma_{0}$ and $\gamma_{0}+\gamma_{1}$, are also equal to zero (i.e., $\gamma_{0}=\gamma_{1}=0$).

To evaluate the power defined in (11), we control the family‐wise error rate at α=0.05 using the Bonferroni method to specify the rejection region C(α). Specifically, we set $C(0.05)=\{(t_{1},t_{2}):t_{1}>z_{0.025}\;\text{or}\;t_{2}>z_{0.025}\}$. We can use more efficient rejection regions by incorporating the correlation between the populations [3, 4]. In the crossover analysis, the proportion of the carryover effect κ was set to 0.0 (no carryover), 0.3 (moderate carryover), and 0.6 (large carryover). We provide numerical results when the marker prevalence is set to p=0.5, although the same tendency was observed for the other values of p in the efficiency comparison between the crossover and parallel‐group analyses (data not shown).

**FIGURE 1:**
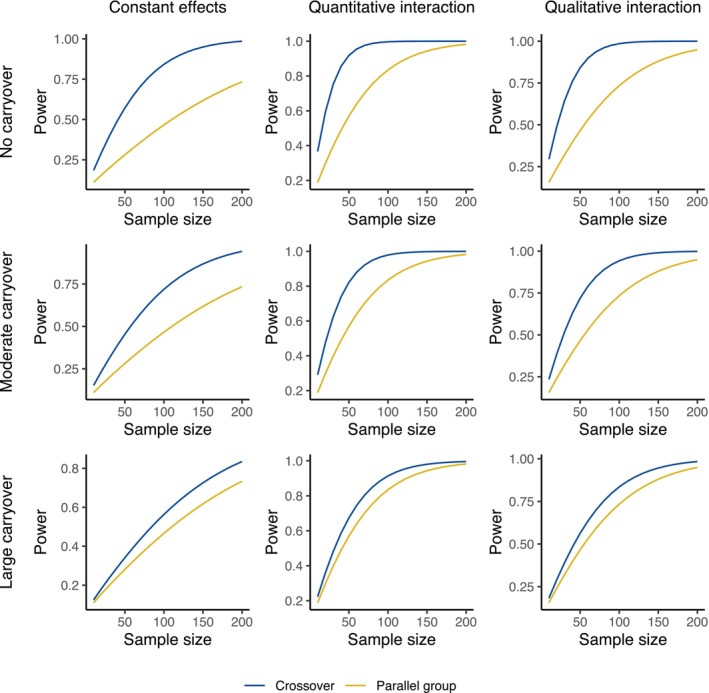
Comparison of the power curve between the crossover and parallel‐group analyses for the three treatment effect scenarios: constant effects ($\Delta_{1}=\Delta_{0}=0.5$; left), quantitative interaction ($\Delta_{1}=1.0,\Delta_{0}=0.5$; center), and qualitative interaction ($\Delta_{1}=1.0,\Delta_{0}=0.0$; right). The results are shown for three levels of carryover effects: no carryover (κ=0.0; top), moderate carryover (κ=0.3; middle), and large carryover (κ=0.6; bottom). The horizontal axis represents the total sample size n, and vertical axis represents the statistical power.

First, we examined the impact of sample size on statistical power for fixed values of the random effects parameters. Figure 1 compares the power curves for the crossover and parallel‐group analyses across the three scenarios and varying values of carryover effects when $\xi_{m}=0,\xi_{t}=0,\rho_{m}=1,$ and $\rho_{t}=1$. Note that we set the means of the two random effects parameters to zero, assuming a preprocessing that subtracts the overall means from the response variable $Y_{ij}$, $Y_{i1}^{*}=Y_{i1}-{\bar{Y}}_{1}$ and $Y_{i2}^{*}=Y_{i2}-\bar{Y_{1}}-(\bar{Y_{2}}-\bar{Y_{1}})=Y_{i2}-\bar{Y_{2}}$, where $\bar{Y_{j}}=\sum_{i=1}^{n}Y_{ij}/n$ (j=1,2) [10].

The results indicate that the crossover analysis consistently achieves higher statistical power than the parallel‐group analysis across all the scenarios, particularly when there are no carryover effects (κ=0.0). The results were consistent for other values of the random effects parameters including non‐zero values for the mean parameters (see Section S1 in the Supporting Informations).

**FIGURE 2:**
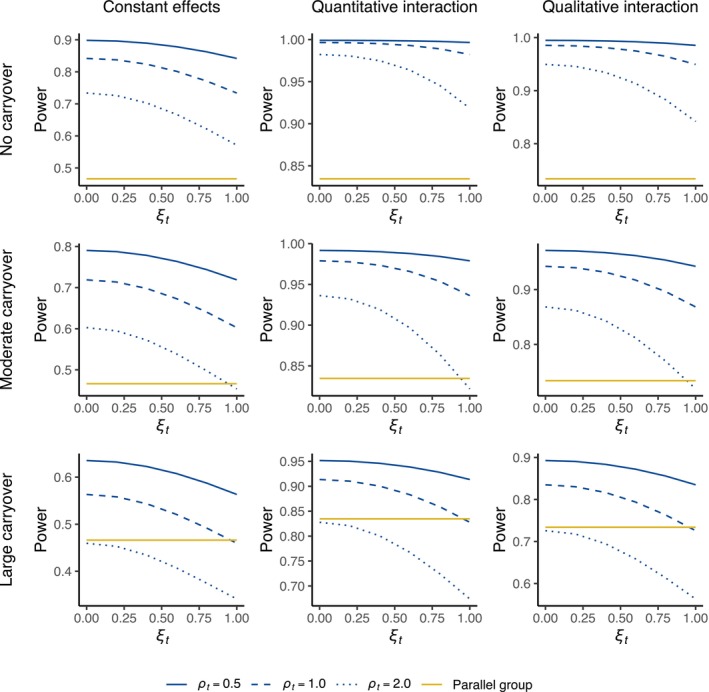
Impact of the parameters of the random effect $t_{i}$ on the power curve of the crossover and parallel‐group analyses. The blue solid line is the power curve in the crossover analysis for the three treatment effect scenarios: constant effects ($\Delta_{1}=\Delta_{0}=0.5$; left), quantitative interaction ($\Delta_{1}=1.0,\Delta_{0}=0.5$; center), and qualitative interaction ($\Delta_{1}=1.0,\Delta_{0}=0.0$; right), with no carryover (κ=0.0; top), moderate carryover (κ=0.3; middle), and large carryover (κ=0.6; bottom). The horizontal axis represents the standardized mean $\xi_{t}$ of the random effect $t_{i}$. The yellow solid line is the power curve in the parallel‐group analysis in the same setting, which does not vary with $\xi_{t}$ and $\rho_{t}$.

**FIGURE 3:**
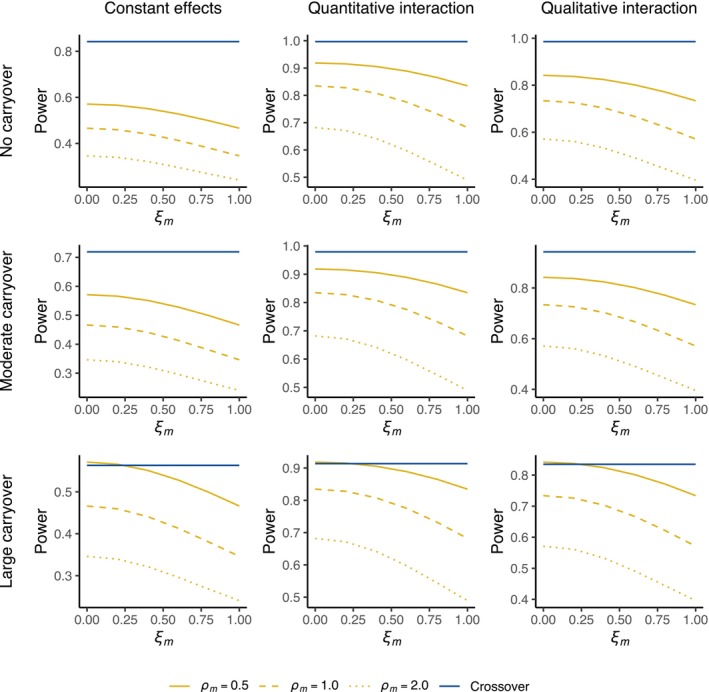
Impact of the parameters of the random effect $m_{i}$ on the power curve of the crossover and parallel‐group analyses. The yellow solid line is the power curve in the parallel‐group analysis for the three treatment effect scenarios: constant effects ($\Delta_{1}=\Delta_{0}=0.5$; left), quantitative interaction ($\Delta_{1}=1.0,\Delta_{0}=0.5$; center), and qualitative interaction ($\Delta_{1}=1.0,\Delta_{0}=0.0$; right), with no carryover (κ=0.0; top), moderate carryover (κ=0.3; middle), and large carryover (κ=0.6; bottom). The horizontal axis represents the standardized mean $\xi_{m}$ of the random effect $m_{i}$. The blue solid line is the power curve in the crossover analysis in the same setting, which does not vary with $\xi_{m}$ and $\rho_{m}$.

Next, we examined the impact of random effects parameters on statistical power. From Figure 2, we can inspect the impact of the standardized mean $\left|\xi_{t}\right|$ and variance ratio $\rho_{t}$ of the random effect $t_{i}$, which affect the performance of the crossover analysis (not the parallel‐group analysis). The figure shows that increases in $\left|\xi_{t}\right|$ and $\rho_{t}$ reduce the statistical power for the crossover analysis owing to the increased variability in the patient responses over time. By contrast, from Figure 3, we can inspect the impact of the standardized mean $\left|\xi_{m}\right|$ and variance ratio $\rho_{m}$ of the random effect $m_{i}$, which affect the performance of the parallel‐group analysis (not the crossover analysis). Here, $\left|\xi_{m}\right|$ and $\rho_{m}$ reduce the statistical power owing to the increased variability between patients.

We also evaluated the required sample size for the crossover and parallel‐group trials under the three scenarios where $\Delta_{1}\geq \Delta_{0}\geq 0$. Table 2 summarizes the required sample sizes to achieve a power of 90% for various values of $\Delta_{1}$ and $\Delta_{0}$ when $\xi_{m}=0,\xi_{t}=0,\rho_{m}=\rho_{t}=1$ and there are no carryover effects. The results indicate that the crossover analysis consistently requires substantially smaller sample sizes compared to the parallel‐group analysis.

**TABLE 2:** Required sample sizes for achieving 90% power for the crossover and parallel‐group analyses under the scenarios with $\Delta_{1}\geq \Delta_{0}\geq 0$ and no carryover effects (κ=0.0).

	Crossover analysis						Parallel‐group analysis					
	$\Delta_{1}$						$\Delta_{1}$					
$\Delta_{0}$	0.0	0.2	0.4	0.6	0.8	1.0	0.0	0.2	0.4	0.6	0.8	1.0
0.0	—	1509	378	168	95	61	—	4023	1006	447	252	161
0.2		755	291	146	86	57		2012	775	388	229	151
0.4			189	113	73	51			503	301	194	134
0.6				84	59	43				224	157	115
0.8					48	37					126	97
1.0						31						81

## 4 Application to a Diabetes Trial

We evaluated the statistical power and required sample sizes on the basis of actual data from a clinical trial in Type 2 diabetes [13]. This study compared two competitive drugs: metformin, a hepatic glucose output inhibitor through pleiotropic effects including AMPK activation, and alogliptin, a dipeptidyl peptidase‐4 inhibitor. The trial enrolled patients aged 20 years or older with HbA1c > 6.5%, who were receiving diet, oral hypoglycemic, or insulin therapy [13]. Metformin reduces glucose levels by suppressing the hepatic glucose output, whereas alogliptin enhances insulin secretion by elevating incretin levels [14]. Of the 84 eligible patients randomized to one of two treatment sequences with two treatment periods, 62 patients completed both treatment periods without dropout and had no missing baseline gene expression data (see Figure S5 in Section S2.1 in Supporting Information for data flow). Of the 62 patients, 31 were in the sequence receiving alogliptin followed by metformin, and the remaining 31 were in the sequence receiving metformin followed by alogliptin. In both periods, patients were administered the study drug for 12 weeks, and the change in HbA1c from baseline to 12 weeks after treatment was measured as the response variable. One reason for focusing on HbA1c as the response variable among multiple endpoints in this trial was due to the biological hypothesis that gene expressions in peripheral blood cells are potentially related to plasma HbA1c levels in Type 2 diabetic patients with metformin therapies [15].

As a predictive marker for metformin efficacy (relative to alogliptin), we considered the baseline expression level of the gene IFNAR1, measured in peripheral blood cells obtained before the start of the first treatment period. IFNAR1 is involved in the expression of interferon, which is thought to act together with inflammatory cytokines to promote insulin resistance [16, 17, 18]. As insulin resistance results in exaggerated hepatic glucose output, metformin may overcome hepatic insulin resistance by suppressing hepatic glucose output independently of insulin action.

The marker‐positive subpopulation was determined in an unsupervised, data‐driven manner on the basis of IFNAR1 level. Specifically, using the k‐means clustering method with k=2, we determined that 29 patients were marker‐positive, corresponding to an estimated marker prevalence of p=0.47. After substituting $Y_{ij}$ in the model in (1) with $Y_{ij}^{*}$, obtained by the location shift of the response variable [10] as discussed in Section 3, we estimated the mixed‐effects model parameters using Bayesian estimation with a non‐informative prior distribution. From the fitted model, the random effect parameter estimates were ${\tilde{\mu }}_{m}=0.014,{\tilde{\sigma }}_{m}^{2}=0.014,{\tilde{\mu }}_{t}=-0.028$, and ${\tilde{\sigma }}_{t}^{2}=0.027$, along with the error variance ${\tilde{\sigma }}_{e}^{2}=0.291$. Using these estimates, we calculated ${\tilde{\xi }}_{m}=0.142,{\tilde{\rho }}_{m}=0.036,{\tilde{\xi }}_{t}=-0.168$, and ${\tilde{\rho }}_{t}=0.093$.

**FIGURE 4:**
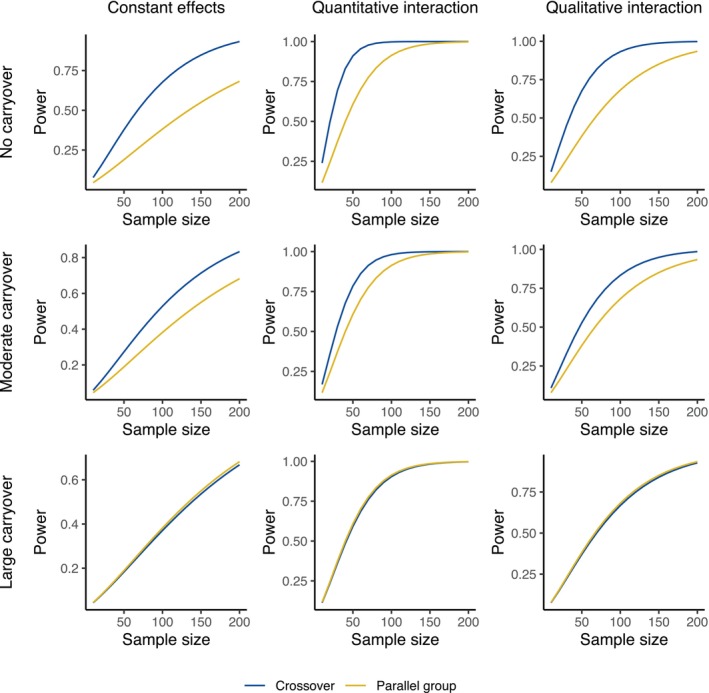
Comparison of the power curve between the crossover and parallel‐group analyses for the three treatment effect scenarios: constant effects ($\Delta_{1}=\Delta_{0}=0.5$; left), quantitative interaction ($\Delta_{1}=1.0,\Delta_{0}=0.5$; center), and qualitative interaction ($\Delta_{1}=1.0,\Delta_{0}=0.0$; right). The results are shown for three levels of carryover effects: no carryover (κ=0.0; top), moderate carryover (κ=0.3; middle), and large carryover (κ=0.6; bottom). The random effects parameters are set to the estimated values from the actual crossover trial data: ${\tilde{\xi }}_{m}=0.142,{\tilde{\rho }}_{m}=0.036,{\tilde{\xi }}_{t}=-0.168$, and ${\tilde{\rho }}_{t}=0.093$. The horizontal axis represents the total sample size n and the vertical axis represents the statistical power.

Figure 4 compares the power curves for the crossover and parallel‐group analyses when the random effects parameters were set to the estimated values ${\tilde{\xi }}_{m}=0.142,{\tilde{\rho }}_{m}=0.036,{\tilde{\xi }}_{t}=-0.168$, and ${\tilde{\rho }}_{t}=0.093$. The results indicate that, under no or moderate carryover effects (κ=0 or 0.3), the crossover analysis consistently provided higher statistical power than the parallel‐group analysis. Even under large carryover effects (κ=0.6), where the advantage of the crossover analysis would be diminished, its performance remained comparable to that of the parallel‐group analysis. With respect to the required sample sizes for achieving 90% power for the same values of the random effects parameters, as shown in Table 3, the crossover analysis consistently required smaller sample sizes compared to the parallel‐group analysis. Note that the results obtained using the original response variable $Y_{ij}$, rather than $Y_{ij}^{*}$ after the location shift, were consistent with those obtained using $Y_{ij}^{*}$, indicating that the crossover analysis achieves higher statistical power and requires smaller sample sizes than the parallel‐group analysis under settings similar to those of the diabetes trial (see Section S2.2 in the Supporting Informations).

**TABLE 3:** Required sample sizes for achieving 90% power for the crossover and parallel‐group analyses under the scenarios with $\Delta_{1}\geq \Delta_{0}\geq 0$ in the application to the diabetes study.

	Crossover analysis						Parallel‐group analysis					
	$\Delta_{1}$						$\Delta_{1}$					
$\Delta_{0}$	0.0	0.2	0.4	0.6	0.8	1.0	0.0	0.2	0.4	0.6	0.8	1.0
0.0	—	1054	264	118	66	43	—	2085	522	232	131	84
0.2		527	203	102	60	40		1043	402	201	119	78
0.4			132	79	51	35			261	156	101	70
0.6				59	42	30				116	82	60
0.8					33	26					66	50
1.0						22						42

## 5 Discussion

This study demonstrated the potential of substantial efficiency gain by the crossover analysis in clinical trials aimed at evaluating treatment efficacy in multiple populations. The proposed crossover analysis using a predictive marker could be useful in many clinical trials, including early‐phase proof‐of‐concept trials for marker‐based treatments and the development and validation of personalized medicine for rare diseases, where sample sizes are inherently limited.

As noted in Section 2.2, the efficiency of the crossover analysis depends on the mean $\mu_{t}$ and variance $\sigma_{t}^{2}$ of the random effect $t_{i}$. If the disease is stable, $\mu_{t}$, which represents the transition in the response variable within patients, is generally not considered to be significant. However, changes in study conditions across treatment periods or carryover effects from the first treatment can affect $\mu_{t}$, emphasizing the importance of careful trial design to mitigate these issues. As for carryover effects, specification of an adequate washout period is important to minimize efficiency loss in the crossover analysis. Meanwhile, the variance $\sigma_{t}^{2}$ is likely to have a limited impact in trials with short study periods; however, it may require attention if patient follow‐up periods, including treatment periods, need to be extended. With these considerations, the crossover analysis can be effectively adopted, especially when efficient use of limited resources is a priority.

Meanwhile, the parallel‐group analysis is influenced by between‐patient variability, captured by the mean $\mu_{m}$ and variance $\sigma_{m}^{2}$ of the random effect $m_{i}$. These factors reflect heterogeneity in patient populations and can be managed, at least partially, through rigorous patient selection criteria. Note that the impact of $\mu_{t}$ and $\mu_{m}$ can be reduced by the location shift of the response variable [10] as discussed in Section 3 before the model fitting.

A potential direction for future research is the integration of the crossover analysis with adaptive clinical trial designs. Interim analyses based on the first‐period data may facilitate flexible adaptations such as reassigning treatments or redefining target populations in the second period. Extension to higher‐order crossover analysis is another interesting direction for separating carryover and period effects from the estimation of treatment effects in multiple populations. Addressing these and other topics in future research can further enhance the utility of the crossover analysis in clinical trials for evaluating multiple populations, thereby contributing to advancements in personalized medicine.

## Funding

This work was supported by the Japan Society for the Promotion of Science (Grant Nos. 16H06299 and 21H04874) and Japan Science and Technology Agency (Grant No. JPMJCR21D3).

## Conflicts of Interest

The authors declare no conflicts of interest.

## Supporting information




**Section S1:** Extended simulation results: Random effects, marker prevalence, and opposing treatment effects.
**Figure S1:** Comparison of the power curve between the crossover and parallel‐group analyses for the three treatment effect scenarios: constant effects ($\Delta_{1}$ = $\Delta_{0}=0.5$; left), quantitative interaction ($\Delta_{1}=1.0$, $\Delta_{0}=0.5$; center), and qualitative interaction ($\Delta_{1}=1.0$, $\Delta_{0}=0.0$; right) with the random effects parameters, $\xi_{m}=1.0$, $\xi_{t}=1.0$, $\rho_{m}=1.0$, and $\rho_{t}=1.0$. The results are shown for three levels of carryover effects: no carryover (κ=0.0; top), moderate carryover (κ=0.3; middle), and large carryover (κ=0.6; bottom). The horizontal axis represents the total sample size n, and the vertical axis represents the statistical power.
**Figure S2:** Comparison of the power curve between the crossover and parallel‐group analyses for the three treatment effect scenarios: constant effects ($\Delta_{1}$ = $\Delta_{0}=0.5$; left), quantitative interaction ($\Delta_{1}=1.0$, $\Delta_{0}=0.5$; center), and qualitative interaction ($\Delta_{1}=1.0$, $\Delta_{0}=0.0$; right) with the random effects parameters, $\xi_{m}=1.0$, $\xi_{t}=0.2$, $\rho_{m}=2.0$, and $\rho_{t}=2.0$. The results are shown for three levels of carryover effects: no carryover (κ=0.0; top), moderate carryover (κ=0.3; middle), and large carryover (κ=0.6; bottom). The horizontal axis represents the total sample size n, and the vertical axis represents the statistical power.
**Figure S3:** Comparison of the power curve between the crossover and parallel‐group analyses for the three treatment effect scenarios: constant effects ($\Delta_{1}$ = $\Delta_{0}=0.5$; left), quantitative interaction ($\Delta_{1}=1.0$, $\Delta_{0}=0.5$; center), and qualitative interaction ($\Delta_{1}=1.0$, $\Delta_{0}=0.0$; right) with the random effects parameters, $\xi_{m}=0.2$, $\xi_{t}=1.0$, $\rho_{m}=0.5$, and $\rho_{t}=2.0$. The results are shown for three levels of carryover effects: no carryover (κ=0.0; top), moderate carryover (κ=0.3; middle), and large carryover (κ=0.6; bottom). The horizontal axis represents the total sample size n, and the vertical axis represents the statistical power.
**Figure S4:** Comparison of the power curve between the crossover and parallel‐group analyses for the scenario with reversed treatment effects between the two subpopulations ($\Delta_{1}=1.0$, $\Delta_{0}=-0.5$) with the random effects parameters, $\xi_{m}=0.0$, $\xi_{t}=0.0$, $\rho_{m}=1.0$, and $\rho_{t}=1.0$. The results are shown for three levels of carryover effects: no carryover (κ=0.0; left), moderate carryover (κ=0.3; center), and large carryover (κ=0.6; right). The horizontal axis represents the total sample size n, and the vertical axis represents the statistical power.
**Section S2.1:** Patient flow for the analysis of treatment effect modification in the diabetes clinical trial.
**Figure S5:** Patient flow diagram for the analysis of treatment effect modification. Of the 84 eligible patients randomized, 62 completed both treatment periods without dropout and had no missing baseline gene expression data. The final analysis set consisted of 31 patients in the sequence receiving alogliptin followed by metformin, and 31 patients in the sequence receiving metformin followed by alogliptin.
**Section S2.2:** Application to a diabetes trial without the location shift of the response variable.
**Figure S6:** Comparison of the power curve between the crossover and parallel‐group analyses for the three treatment effect scenarios: constant effects ($\Delta_{1}$ = $\Delta_{0}=0.5$; left), quantitative interaction ($\Delta_{1}=1.0$, $\Delta_{0}=0.5$; center), and qualitative interaction ($\Delta_{1}=1.0$, $\Delta_{0}=0.0$; right). The results are shown for three levels of carryover effects: no carryover (κ=0.0; top), moderate carryover (κ=0.3; middle), and large carryover (κ=0.6; bottom). The random effects parameters are set to the estimated values from the actual crossover trial data, ${\tilde{\xi }}_{m}=-4.371,{\tilde{\rho }}_{m}=0.036,{\tilde{\xi }}_{t}=2.982$, and ${\tilde{\rho }}_{t}=0.093$. The horizontal axis represents the total sample size n, and the vertical axis represents the statistical power.
**Table S1:** Required sample sizes for achieving 90% power for the crossover and parallel‐group analyses under the scenarios with $\Delta_{1}\geq \Delta_{0}\geq 0$ in the application to the diabetes study without the location shift of the response variable.
**Section S3.1:** Variances of $R_{i}$ and $Z_{i}$.
**Section S3.2:** Derivation of the estimators for the treatment effect parameters.
**Section S3.3:** Equivalence conditions of the two estimators ${\hat{\theta }}_{j}^{*}$ and ${\hat{\theta }}_{j}$.
**Section S3.4:** Covariance of test statistics.
**Section S3.5:** Extension to unequal allocation ratios.
**Section S3.6:** Correspondence with conventional crossover design notation.

## Data Availability

The data that support the findings of this study are available from the corresponding author upon reasonable request.
