# Supplementary material for: Efficiency Enhancement in Testing Treatment Efficacy Across Multiple Populations Using Treatment Crossover Data
Source: Stat Med. 2026 Jul 15;45(15-17):e70651. doi: 10.1002/sim.70651 (PMC13371832; doi:10.1002/sim.70651)
Supplement: Supplementary file 1 — Section S1: Extended simulation results: Random effects, marker prevalence, and opposing treatment effects. Figure S1: Comparison of the power curve between the crossover and parallel‐group analyses for the three treatment effect scenarios: constant effects (Δ1 = Δ0=0.5; left), quantitative interaction (Δ1=1.0, Δ0=0.5; center), and qualitative interaction (Δ1=1.0, Δ0=0.0; right) with the random effects parameters, ξm=1.0, ξt=1.0, ρm=1.0, and ρt=1.0. The results are shown for three levels of carryover effects: no carryover (κ=0.0; top), moderate carryover (κ=0.3; middle), and large carryover (κ=0.6; bottom). The horizontal axis represents the total sample size n, and the vertical axis represents the statistical power. Figure S2: Comparison of the power curve between the crossover and parallel‐group analyses for the three treatment effect scenarios: constant effects (Δ1 = Δ0=0.5; left), quantitative interaction (Δ1=1.0, Δ0=0.5; center), and qualitative interaction (Δ1=1.0, Δ0=0.0; right) with the random effects parameters, ξm=1.0, ξt=0.2, ρm=2.0, and ρt=2.0. The results are shown for three levels of carryover effects: no carryover (κ=0.0; top), moderate carryover (κ=0.3; middle), and large carryover (κ=0.6; bottom). The horizontal axis represents the total sample size n, and the vertical axis represents the statistical power. Figure S3: Comparison of the power curve between the crossover and parallel‐group analyses for the three treatment effect scenarios: constant effects (Δ1 = Δ0=0.5; left), quantitative interaction (Δ1=1.0, Δ0=0.5; center), and qualitative interaction (Δ1=1.0, Δ0=0.0; right) with the random effects parameters, ξm=0.2, ξt=1.0, ρm=0.5, and ρt=2.0. The results are shown for three levels of carryover effects: no carryover (κ=0.0; top), moderate carryover (κ=0.3; middle), and large carryover (κ=0.6; bottom). The horizontal axis represents the total sample size n, and the vertical axis represents the statistical power. Figure S4: Comparison of the power [file SIM-45-0-s001.pdf]

Supplementary Material: Efficiency enhancement in testing treatment efficacy across multiple populations using treatment crossover data

Ryo Emoto[1,*], Kiyoaki Ishii[2,3], Toshinari Takamura[2], Shigeyuki Matsui[1,4]

[1]Department of Biostatistics and Data Science, School of Public Health, Graduate School of Medicine, Kyoto University, Kyoto, Japan

[2]Department of Endocrinology and Metabolism, Kanazawa University Graduate School of Medical Sciences, Ishikawa, Japan

[3]Department of Musculoskeletal Disease, National Center for Geriatrics and Gerontology, Aichi, Japan

[4]Department of Interdisciplinary Statistical Mathematics, The Institute of Statistical Mathematics, Tokyo, Japan

Contents

S1 Extended simulation results: random effects, marker prevalence, and opposing treatment effects **2**

S2 Supplementary results for the diabetes clinical trial **8**

S2.1 Patient flow for the analysis of treatment effect modification in the diabetes clinical trial . . . 8

S2.2 Application to a diabetes trial without the location shift of the response variable . . . 8

S3 Mathematical Derivations and Extensions **10**

S3.1 Variances of R_i and Z_i . . . 10

S3.2 Derivation of the estimators for the treatment effect parameters . . . 11

S3.3 Equivalence conditions of the two estimators $\hat{\theta}_j^*$ and $\hat{\theta}_j$ . . . 14

S3.4 Covariance of test statistics . . . 15

S3.5 Extension to unequal allocation ratios . . . 16

S3.6 Correspondence with conventional crossover design notation . . . 17

S1 Extended simulation results: random effects, marker prevalence, and opposing treatment effects

This section presents simulation results for other values of the random effects parameters and reversed treatment effects between the marker-positive population and its complementary subpopulation ($\Delta_1 > 0, \Delta_0 < 0$).

We have evaluated the statistical power of the crossover and parallel-group analyses for a wide variety of values of the random effects parameters ξ_m, ρ_m, ξ_t, and ρ_t. In this section, we present some typical results to highlight the main features for the method comparison by investigating the impact of non-zero values for the mean parameters ξ_m and ξ_t and different values of the variance parameters ρ_m and ρ_t. Specifically, we considered the following three configurations:

1. Non-zero means for random effects parameters: $\xi_m = 1.0, \xi_t = 1.0, \rho_m = 1.0$, and $\rho_t = 1.0$.
2. Large between-patient variability and small within-patient variability: $\xi_m = 1.0$, $\xi_t = 0.2$, $\rho_m = 2.0$, and $\rho_t = 0.5$.
3. Small between-patient variability and large within-patient variability: $\xi_m = 0.2$, $\xi_t = 1.0$, $\rho_m = 0.5$, and $\rho_t = 2.0$.

Figures S1 and S2 show the results for the first two configurations. In these settings, the crossover analysis consistently achieves higher statistical power than the parallel-group analysis across all three treatment effect scenarios when the carryover effects are null ($\kappa = 0.0$). As the carryover effects increase ($\kappa = 0.6$), the power advantage of the crossover analysis diminishes but remains evident, especially in Scenarios 2 and 3, where the treatment effect is more pronounced in the marker-positive subpopulation. These results align closely with the trends observed in the main text (Figure 1), indicating consistent efficiency gain from the crossover analysis even if between-patient variability dominates or when the random effect means are non-zero. By contrast, Figure S3 shows

the results for the third configuration. In this extreme scenario, the crossover analysis does not consistently outperform the parallel-group analysis. Under null carryover effects ($\kappa = 0.0$), both analyses achieve comparable statistical power. However, as the carryover effects increase ($\kappa = 0.6$), the parallel-group analysis exhibits higher power than the crossover analysis. This reduction in the efficiency of the crossover analysis is attributed to the large within-patient variability ρ_t, which diminishes the benefits from within-patient comparisons. This result emphasizes the importance of considering the size of within-patient variability relative to between-patient variability in the target patient population when selecting the trial design. Except for this extreme case, the crossover design is generally more efficient than the parallel-group analysis.

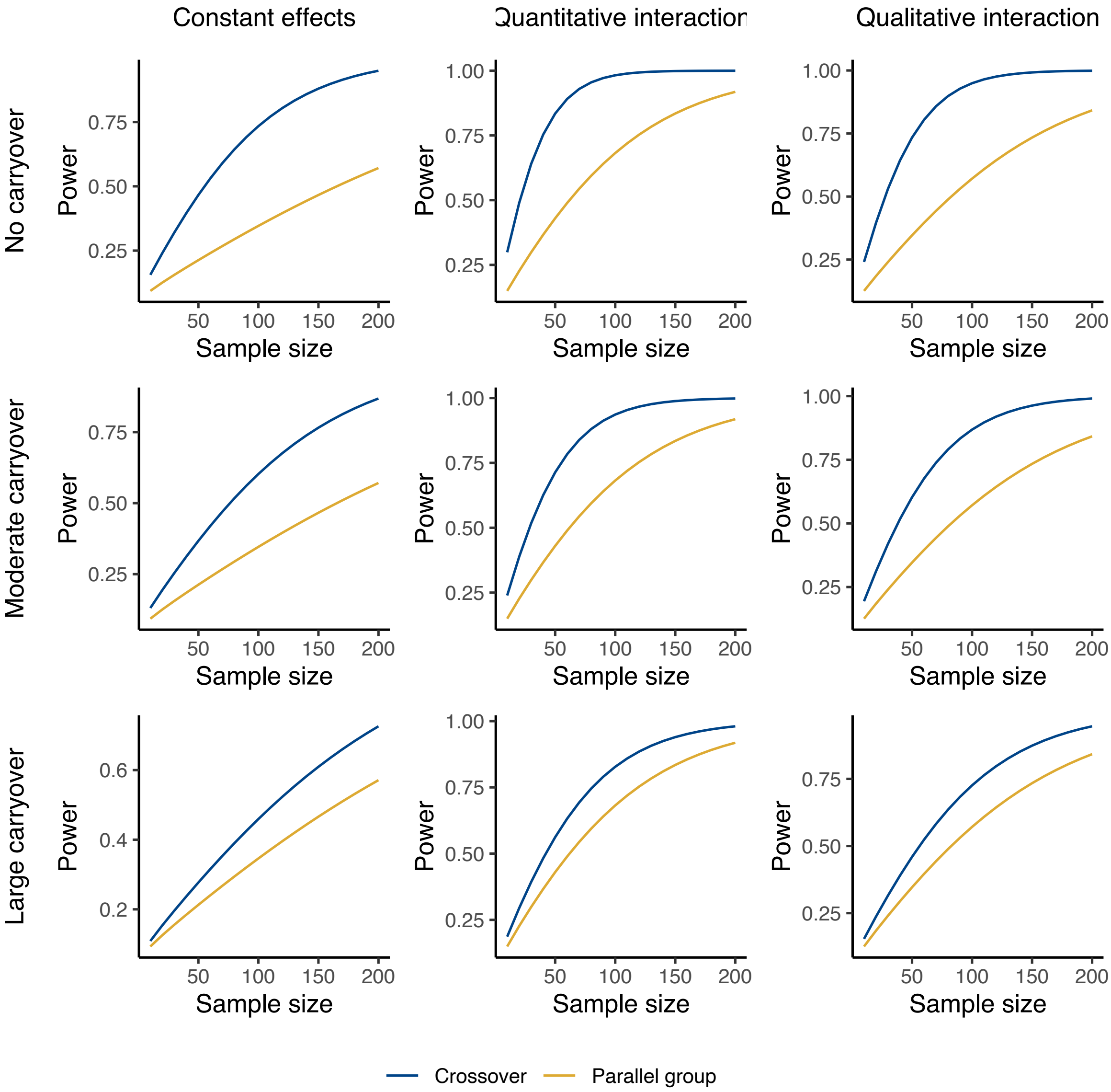

Figure S1: Comparison of the power curve between the crossover and parallel-group analyses for the three treatment effect scenarios: constant effects ($\Delta_1 = \Delta_0 = 0.5$; left), quantitative interaction ($\Delta_1 = 1.0, \Delta_0 = 0.5$; center), and qualitative interaction ($\Delta_1 = 1.0, \Delta_0 = 0.0$; right) with the random effects parameters, $\xi_m = 1.0, \xi_t = 1.0, \rho_m = 1.0$, and $\rho_t = 1.0$. The results are shown for three levels of carryover effects: no carryover ($\kappa = 0.0$; top), moderate carryover ($\kappa = 0.3$; middle), and large carryover ($\kappa = 0.6$; bottom). The horizontal axis represents the total sample size n, and the vertical axis represents the statistical power.

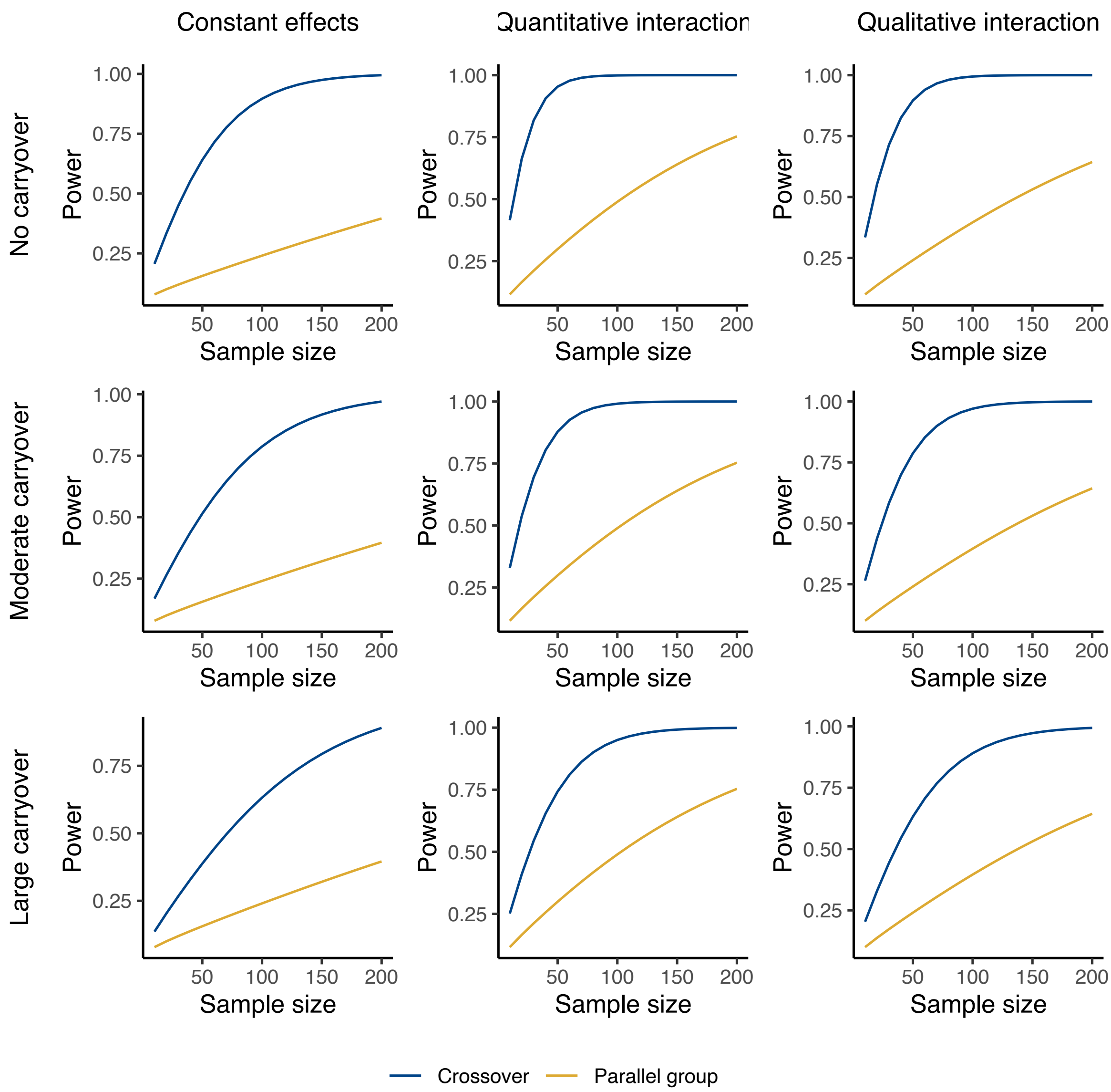

Figure S2: Comparison of the power curve between the crossover and parallel-group analyses for the three treatment effect scenarios: constant effects ($\Delta_1 = \Delta_0 = 0.5$; left), quantitative interaction ($\Delta_1 = 1.0, \Delta_0 = 0.5$; center), and qualitative interaction ($\Delta_1 = 1.0, \Delta_0 = 0.0$; right) with the random effects parameters, $\xi_m = 1.0, \xi_t = 0.2, \rho_m = 2.0$, and $\rho_t = 0.5$. The results are shown for three levels of carryover effects: no carryover ($\kappa = 0.0$; top), moderate carryover ($\kappa = 0.3$; middle), and large carryover ($\kappa = 0.6$; bottom). The horizontal axis represents the total sample size n, and the vertical axis represents the statistical power.

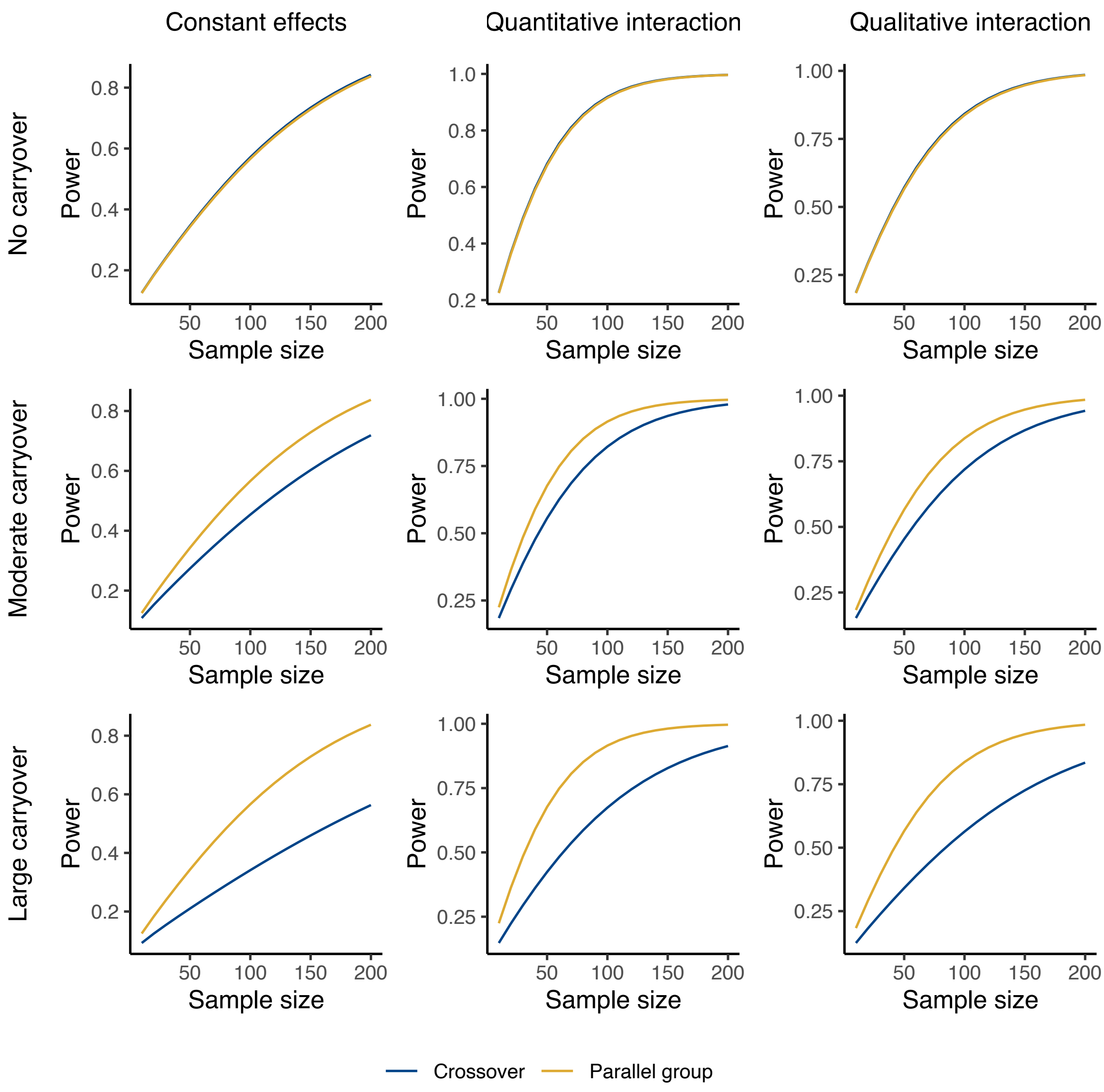

Figure S3: Comparison of the power curve between the crossover and parallel-group analyses for the three treatment effect scenarios: constant effects ($\Delta_1 = \Delta_0 = 0.5$; left), quantitative interaction ($\Delta_1 = 1.0, \Delta_0 = 0.5$; center), and qualitative interaction ($\Delta_1 = 1.0, \Delta_0 = 0.0$; right) with the random effects parameters, $\xi_m = 0.2, \xi_t = 1.0, \rho_m = 0.5$, and $\rho_t = 2.0$. The results are shown for three levels of carryover effects: no carryover ($\kappa = 0.0$; top), moderate carryover ($\kappa = 0.3$; middle), and large carryover ($\kappa = 0.6$; bottom). The horizontal axis represents the total sample size n, and the vertical axis represents the statistical power.

Finally, we investigated scenarios with reversed treatment effects between the marker-positive population and its complementary subpopulation ($\Delta_1 > 0, \Delta_0 < 0$). Importantly, even when $\Delta_0 < 0$, which corresponds to a null hypothesis where the control is superior

in the complementary subpopulation, the hypothesis tests in the marker-positive and overall populations remained one-sided tests of treatment effects for the superiority of the experimental drug over the control. Across all scenarios with varying sizes of the carryover effects, the crossover analysis provided greater power than the parallel-group analysis (see Figure S4).

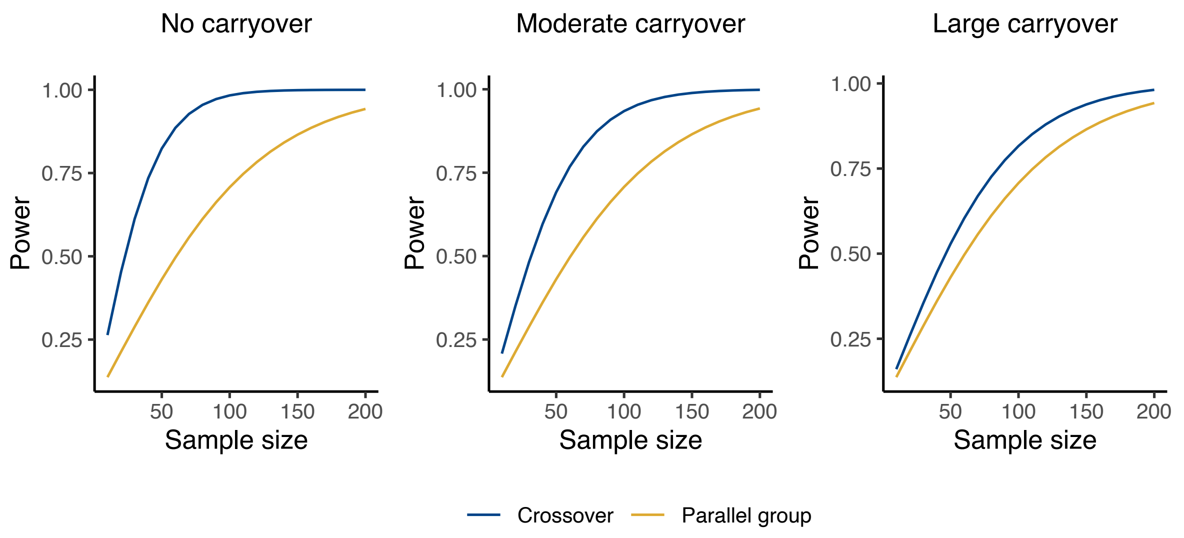

Figure S4: Comparison of the power curve between the crossover and parallel-group analyses for the scenario with reversed treatment effects between the two subpopulations ($\Delta_1 = 1.0, \Delta_0 = -0.5$) with the random effects parameters, $\xi_m = 0.0, \xi_t = 0.0, \rho_m = 1.0$, and $\rho_t = 1.0$. The results are shown for three levels of carryover effects: no carryover ($\kappa = 0.0$; left), moderate carryover ($\kappa = 0.3$; center), and large carryover ($\kappa = 0.6$; right). The horizontal axis represents the total sample size n and the vertical axis represents the statistical power.

S2 Supplementary results for the diabetes clinical trial

S2.1 Patient flow for the analysis of treatment effect modification in the diabetes clinical trial

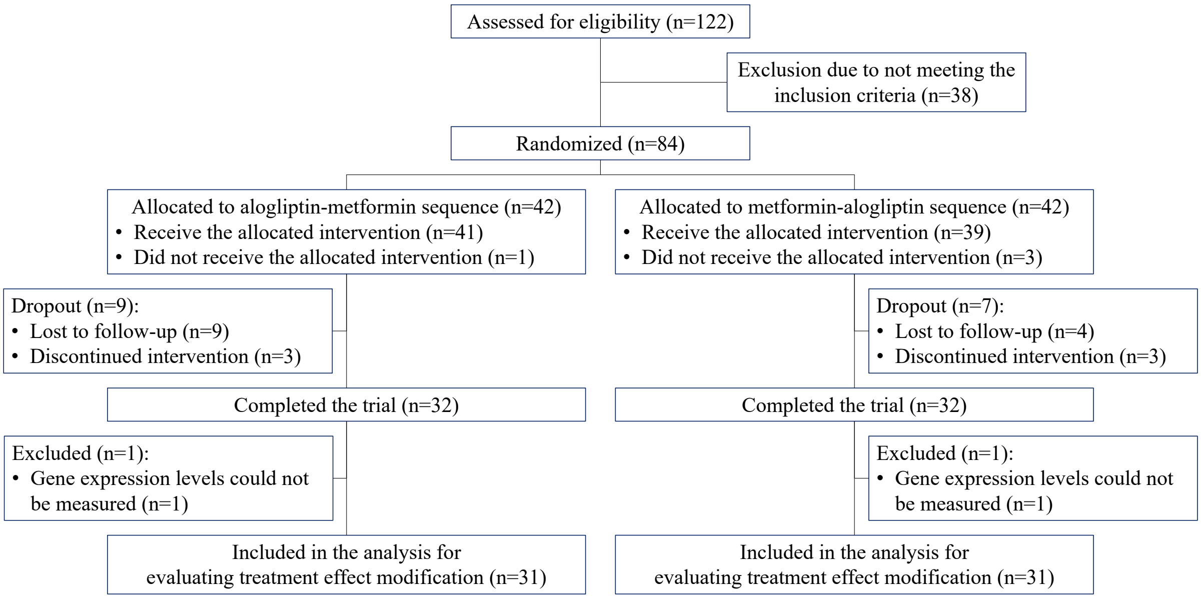

Figure S5: Patient flow diagram for the analysis of treatment effect modification. Of the 84 eligible patients randomized, 62 completed both treatment periods without dropout and had no missing baseline gene expression data. The final analysis set consisted of 31 patients in the sequence receiving alogliptin followed by metformin, and 31 patients in the sequence receiving metformin followed by alogliptin.

S2.2 Application to a diabetes trial without the location shift of the response variable

We also evaluated the statistical power and required sample sizes on the basis of actual data from a clinical trial in type 2 diabetes without location shift of the response variable, where the original response variable Y_{ij} was used instead of Y^*_{ij}. Figure S6 compares the power curves for the crossover and parallel-group analyses when the random effects parameters were set to the estimated values $\tilde{\xi}_m = -4.371, \tilde{\rho}_m = 0.036, \tilde{\xi}_t = 2.982$ and $\tilde{\rho}_t = 0.093$. The results indicate that the crossover analysis provided higher statistical power than the parallel-group analysis. With respect to the required sample sizes for

achieving 90% power for the same values of the random effects parameters, as shown in Table 3, the crossover analysis consistently required smaller sample sizes compared to the parallel-group analysis.

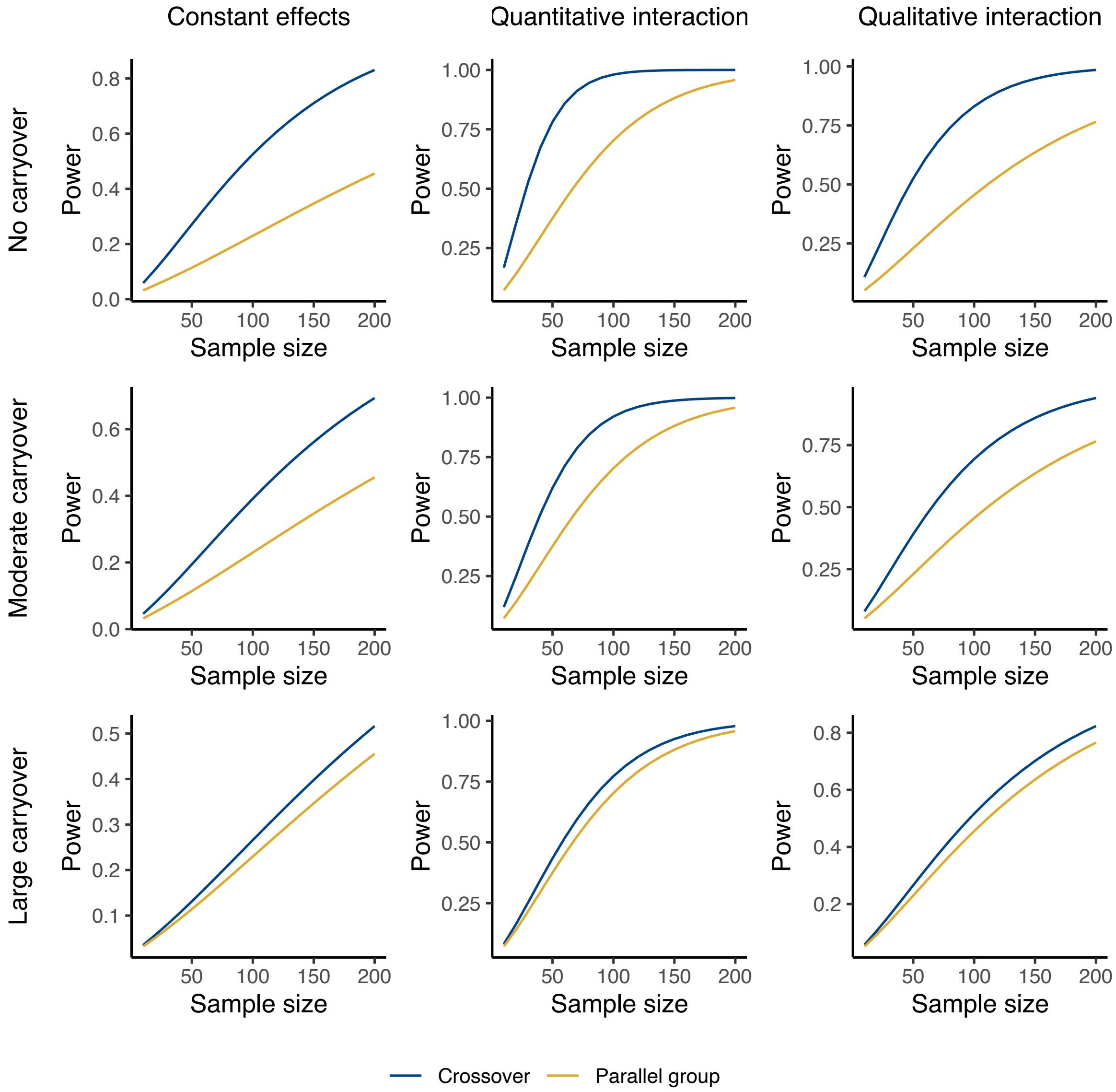

Figure S6: Comparison of the power curve between the crossover and parallel-group analyses for the three treatment effect scenarios: constant effects ($\Delta_1 = \Delta_0 = 0.5$; left), quantitative interaction ($\Delta_1 = 1.0, \Delta_0 = 0.5$; center), and qualitative interaction ($\Delta_1 = 1.0, \Delta_0 = 0.0$; right). The results are shown for three levels of carryover effects: no carryover ($\kappa = 0.0$; top), moderate carryover ($\kappa = 0.3$; middle), and large carryover ($\kappa = 0.6$; bottom). The random effects parameters are set to the estimated values from the actual crossover trial data, $\tilde{\xi}_m = -4.371, \tilde{\rho}_m = 0.036, \tilde{\xi}_t = 2.982$, and $\tilde{\rho}_t = 0.093$. The horizontal axis represents the total sample size n, and the vertical axis represents the statistical power.

Table S1: Required sample sizes for achieving 90% power for the crossover and parallel-group analyses under the scenarios with $\Delta_1 \geq \Delta_0 \geq 0$ in the application to the diabetes study without the location shift of the response variable.

Δ_0 \ Δ_1	Crossover analysis						Parallel-group analysis					
	0.0	0.2	0.4	0.6	0.8	1.0	0.0	0.2	0.4	0.6	0.8	1.0
0.0	–	1469	368	164	92	59	–	3456	864	384	216	139
0.2		735	283	142	84	55		1728	666	333	197	129
0.4			184	110	71	49			432	258	167	115
0.6				82	58	42				192	135	99
0.8					46	36					108	83
1.0						30						70

S3 Mathematical Derivations and Extensions

S3.1 Variances of R_i and Z_i

As introduced in (2) in Section 2.2, R_i is the transformed response variable for within-patient comparisons in the crossover analysis, $R_i = G_i(Y_{i1} - Y_{i2})$. Given that G_i and $(t_i - e_{i1} + e_{i2})$ are independent under random allocation of treatment sequences, we derive the variance of R_i as follows:

$$
\begin{aligned}
\mathrm{Var}(R_i) &= \mathrm{Var}(\epsilon_i) = \mathrm{Var}\left[-G_i(t_i - e_{i1} + e_{i2})\right] \\
&= \mathrm{E}[G_i^2(t_i - e_{i1} + e_{i2})^2] - \mathrm{E}[-G_i(t_i - e_{i1} + e_{i2})]^2 \\
&= \mathrm{E}[t_i^2] + \mathrm{E}[e_{i1}^2] + \mathrm{E}[e_{i2}]^2 \\
&= \mu_t^2 + \sigma_t^2 + 2\sigma_e^2,
\end{aligned}
$$

and covariance $\mathrm{Cov}(R_i, R_{i'}) = 0$ for $i \neq i'$.

Similarly, as introduced in (5) in Section 2.3, Z_i is the transformation defined as $Z_i = 2Y_{i1}A_{i1}$. Under random allocation of treatment in the first period, the variance of

Z_i can be derived as follows:

$$\begin{aligned}
\mathrm{Var}(Z_i) &= \mathrm{Var}(\epsilon_i) = 4\mathrm{Var}\left[A_{i1}(m_i + e_{i1})\right] \\
&= 4\{\mathrm{E}[A_{i1}^2(m_i + e_{i1})^2] - \mathrm{E}[A_{i1}(m_i + e_{i1})]^2\} \\
&= 4\mathrm{E}[m_i^2 + e_{i1}^2] \\
&= 4(\mu_m^2 + \sigma_m^2 + \sigma_e^2),
\end{aligned}$$

and covariance $\mathrm{Cov}(Z_i, Z_{i'}) = 0$ for $i \neq i'$.

S3.2 Derivation of the estimators for the treatment effect parameters

As defined in Section 2.1, δ_1 and δ_2 represent the average treatment effects in the marker-defined subpopulation and the overall population, respectively. Let $\hat{\boldsymbol{\theta}} = (\hat{\theta}_0, \hat{\theta}_1)^T$ be the estimator of $\boldsymbol{\theta} = (\theta_0, \theta_1)^T$; the estimator of the average treatment effects in the marker-defined subpopulation and the overall population, $\boldsymbol{\delta} = (\delta_1, \delta_2)^T$, can be expressed as follows:

$$\hat{\boldsymbol{\delta}} = \begin{pmatrix} 1 & 1 \\ 1 & p \end{pmatrix} \hat{\boldsymbol{\theta}}.$$

In the crossover analysis, we consider the estimator for $\boldsymbol{\psi} = (\psi_0, \psi_1)^T$ with no carryover effects $\boldsymbol{\gamma} = (\gamma_0, \gamma_1)^T = \mathbf{0}$ as the estimator of $\boldsymbol{\theta} = (\theta_0, \theta_1)^T$. When a least-squares method is used, the estimator $\hat{\boldsymbol{\psi}} = (\hat{\psi}_0, \hat{\psi}_1)^T$ can be expressed as follows:

$$\hat{\boldsymbol{\psi}} = (X^T X)^{-1} X^T \boldsymbol{R}$$

where $\boldsymbol{R} = (R_1, \dots, R_n)^T$ and

$$X = \begin{pmatrix} 1 & x_1 \\ 1 & x_2 \\ \vdots & \vdots \\ 1 & x_n \end{pmatrix}.$$

Here, since $\sum_{i=1}^n x_i = \sum_{i=1}^n x_i^2 = np$, the inverse of $X^T X$ can be expressed as follows:

$$\begin{aligned}
(X^T X)^{-1} &= \begin{pmatrix} n & \sum_{i=1}^n x_i \\ \sum_{i=1}^n x_i & \sum_{i=1}^n x_i^2 \end{pmatrix}^{-1} \\
&= \left\{ n \begin{pmatrix} 1 & p \\ p & p \end{pmatrix} \right\}^{-1} \\
&= \frac{1}{np(1-p)} \begin{pmatrix} p & -p \\ -p & 1 \end{pmatrix}.
\end{aligned}$$

Then, the estimator $\hat{\boldsymbol{\delta}}^{\mathrm{CO}} = (\hat{\delta}_1^{\mathrm{CO}}, \hat{\delta}_2^{\mathrm{CO}})^T$ can be expressed as follows:

$$
\begin{aligned}
\hat{\boldsymbol{\delta}}^{\mathrm{CO}} &= \begin{pmatrix} 1 & 1 \\ 1 & p \end{pmatrix} \hat{\boldsymbol{\psi}} \\
&= \begin{pmatrix} 1 & 1 \\ 1 & p \end{pmatrix} (X^T X)^{-1} X^T \boldsymbol{R} \\
&= \frac{1}{np(1-p)} \begin{pmatrix} 1 & 1 \\ 1 & p \end{pmatrix} \begin{pmatrix} p & -p \\ -p & 1 \end{pmatrix} \begin{pmatrix} \sum_{i=1}^n R_i \\ \sum_{i=1}^n x_i R_i \end{pmatrix} \\
&= \frac{1}{np(1-p)} \begin{pmatrix} 0 & 1-p \\ p(1-p) & 0 \end{pmatrix} \begin{pmatrix} \sum_{i=1}^n R_i \\ \sum_{i=1}^n x_i R_i \end{pmatrix} \\
&= \begin{pmatrix} \frac{1}{np} \sum_{i=1}^n x_i R_i \\ \frac{1}{n} \sum_{i=1}^n R_i \end{pmatrix}.
\end{aligned}
$$

In the parallel-group analysis, the estimator $\hat{\boldsymbol{\theta}} = (\hat{\theta}_0, \hat{\theta}_1)^T$ using a least-squares method can be expressed as follows:

$$\hat{\boldsymbol{\theta}} = (X^T X)^{-1} X^T \boldsymbol{Z}$$

where $\boldsymbol{Z} = (Z_1, \ldots, Z_n)^T$. Similar to the case of the crossover analysis, the estimator

$\hat{\boldsymbol{\delta}}^{\mathrm{PG}} = (\hat{\delta}_1^{\mathrm{PG}}, \hat{\delta}_2^{\mathrm{PG}})^T$ can be expressed as follows:

$$\begin{aligned}
\hat{\boldsymbol{\delta}}^{\mathrm{PG}} &= \begin{pmatrix} 1 & 1 \\ 1 & p \end{pmatrix} \hat{\boldsymbol{\theta}} \\
&= \frac{1}{np(1-p)} \begin{pmatrix} 1 & 1 \\ 1 & p \end{pmatrix} \begin{pmatrix} p & -p \\ -p & 1 \end{pmatrix} \begin{pmatrix} \sum_{i=1}^n Z_i \\ \sum_{i=1}^n x_i Z_i \end{pmatrix} \\
&= \begin{pmatrix} \dfrac{1}{np} \sum_{i=1}^n x_i Z_i \\ \dfrac{1}{n} \sum_{i=1}^n Z_i \end{pmatrix}.
\end{aligned}$$

S3.3 Equivalence conditions of the two estimators $\hat{\theta}_j^*$ and $\hat{\theta}_j$

First, we consider the case of ordinal least-squares estimation for models $R_i = \psi(x_i) + \varepsilon_i, S_i = \gamma_0 + \gamma_1 x_i + \varepsilon_i'$, and $Z_i = \theta_0 + \theta_1 x_i + \epsilon_i, (i = 1, \ldots, n)$ to obtain estimators $\hat{\boldsymbol{\psi}} = (\hat{\psi}_0, \hat{\psi}_1)^T, \hat{\boldsymbol{\gamma}} = (\hat{\gamma}_0, \hat{\gamma}_1)^T$, and $\hat{\boldsymbol{\theta}} = (\hat{\theta}_0, \hat{\theta}_1)^T$, respectively, as defined in the main text. Here, the estimators for regression coefficients (including intercept terms) are given by $\hat{\boldsymbol{\psi}} = P\boldsymbol{R}$, $\hat{\boldsymbol{\gamma}} = P\boldsymbol{S}$, and $\hat{\boldsymbol{\theta}} = P\boldsymbol{Z}$, where $P = (X^T X)^{-1} X^T, \boldsymbol{R} = (R_1, \ldots, R_n)^T$, $\boldsymbol{S} = (S_1, \ldots, S_n)^T$, and $\boldsymbol{Z} = (Z_1, \ldots, Z_n)^T$. As defined in the main text, $R_i = G_i(Y_{i1} - Y_{i2})$, $S_i = -2G_i(Y_{i1} + Y_{i2})$, and $Z_i = 2A_{i1}Y_{i1}$ $(i = 1, \ldots, n)$. In the crossover analysis with the estimation of carryover effects, the estimator of $\boldsymbol{\theta} = (\theta_0, \theta_1)^T$ can be expressed as follows:

$$\begin{aligned}
\hat{\boldsymbol{\theta}}^* &= \hat{\boldsymbol{\psi}} - \hat{\boldsymbol{\gamma}}/2 \\
&= P(\boldsymbol{R} - \boldsymbol{S}/2) \\
&= P\boldsymbol{Z} = \hat{\boldsymbol{\theta}},
\end{aligned}$$

because $A_{i1} = G_i$.

We note that the condition for equivalence of $\hat{\boldsymbol{\theta}}^*$ and $\hat{\boldsymbol{\theta}}$ is the use of the same projection matrix P for all the three estimators $\hat{\boldsymbol{\psi}}$, $\hat{\boldsymbol{\gamma}}$, and $\hat{\boldsymbol{\theta}}$. This condition will be held for other projection matrices for P, such as $(X^T W X)^{-1} X^T W$ in weighted least-squares estimators

and $(X^TX+\lambda I_K)^{-1}X^T$ in ridge regression estimators with a fixed penalty parameter λ, where W is a weight matrix of size $n \times n$ and I_K is the identity matrix of size $K \times K$.

S3.4 Covariance of test statistics

The covariance of the test statistics T_1^{CO} and T_2^{CO} in Section 2.4 can be expressed by the following equation:

$$\begin{aligned}
\mathrm{Cov}(T_1^{\mathrm{CO}}, T_2^{\mathrm{CO}}) &= \mathrm{Cov}\left(\hat{\delta}_1^{\mathrm{CO}}/\sqrt{\mathrm{Var}(\hat{\delta}_1^{\mathrm{CO}})}, \hat{\delta}_2^{\mathrm{CO}}/\sqrt{\mathrm{Var}(\hat{\tau}_2^{\mathrm{CO}})}\right) \\
&= \frac{1}{\sqrt{\mathrm{Var}(\varepsilon_i)^2/(n^2p)}}\mathrm{Cov}\left(\frac{1}{np}\sum_{i=1}^{n} x_iR_i, \frac{1}{n}\sum_{i=1}^{n} R_i\right) \\
&= \frac{n\sqrt{p}}{\mathrm{Var}(\varepsilon_i)}\frac{1}{n^2p}\sum_{i\in\{i:x_i=1\}} \mathrm{Var}(R_i) \\
&= \frac{1}{n\sqrt{p}\mathrm{Var}(\varepsilon_i)}\{np\mathrm{Var}(\varepsilon_i)\} \\
&= \sqrt{p}.
\end{aligned}$$

Similarly, the covariance of test statistics T_1^{PG} and T_2^{PG} in Section 2.4 can be expressed by the following equation:

$$\begin{aligned}
\mathrm{Cov}(T_1^{\mathrm{PG}}, T_2^{\mathrm{PG}}) &= \frac{1}{\sqrt{\mathrm{Var}(\epsilon_i)^2/(n^2p)}}\mathrm{Cov}\left(\frac{1}{np}\sum_{i=1}^{n} x_iZ_i, \frac{1}{n}\sum_{i=1}^{n} Z_i\right) \\
&= \frac{n\sqrt{p}}{\mathrm{Var}(\epsilon_i)}\frac{1}{n^2p}\sum_{i\in\{i:x_i=1\}} \mathrm{Var}(Z_i) \\
&= \frac{1}{n\sqrt{p}\mathrm{Var}(\epsilon_i)}\{np\mathrm{Var}(\epsilon_i)\} \\
&= \sqrt{p}.
\end{aligned}$$

S3.5 Extension to unequal allocation ratios

In Sections 2.2 and 2.3 of the main text, we assumed equal allocation ratios. Here, we demonstrate that the proposed estimators can be extended to unequal allocation ratios by applying appropriate transformations.

Crossover analysis with unequal allocation ratios

Let the allocation ratio to the sequences be $q : (1 - q)$, where $q = \Pr(G_i = 1)$ and consequently $\Pr(G_i = -1) = 1 - q$. We introduce a transformed sequence variable G'_i:

$$G'_i = \frac{G_i}{2qG_i + (1 - G_i)} = \begin{cases} \dfrac{1}{2q} & \text{if } G_i = 1, \\ -\dfrac{1}{2(1-q)} & \text{if } G_i = -1. \end{cases} \tag{S.1}$$

Using this, we define the transformed response for the crossover analysis as $R'_i = G'_i(Y_{i1} - Y_{i2})$. The expectation of this transformed variable is:

$$\mathrm{E}[R'_i] = \mathrm{E}[G'_i G_i]\psi(x_i) - \mathrm{E}[G'_i]\mathrm{E}[t_i - e_{i1} + e_{i2}] = \psi(x_i), \tag{S.2}$$

since $\mathrm{E}[G'_i] = 0$ and $\mathrm{E}[G'_i G_i] = 1$. When $q = 1/2$, this reduces to the original estimator.

While the transformation R'_i provides an unbiased estimator of $\psi(x_i)$, the variance of R'_i under unequal allocation ($q \neq 1/2$) is more complex and depends on the treatment effect $\psi(x_i)$. Using the properties $\mathrm{E}[G'^2_i] = \dfrac{1}{4q(1-q)}$, $\mathrm{E}[G'^2_i G_i] = \dfrac{1-2q}{4q(1-q)}$, and the independence between G_i and (t_i, e_{i1}, e_{i2}), the variance can be derived as:

$$\mathrm{Var}(R'_i) = \left\{\frac{1}{4q(1-q)} - 1\right\}\psi(x_i)^2 - \frac{(1-2q)\mu_t}{2q(1-q)}\psi(x_i) + \frac{\sigma_t^2 + \mu_t^2 + 2\sigma_e^2}{4q(1-q)}$$

When $q = 1/2$, this expression simplifies to $\mathrm{Var}(R'_i) = \sigma_t^2 + \mu_t^2 + 2\sigma_e^2$, which is constant and independent of the treatment effect $\psi(x_i)$ (as discussed in the main text). However, for $q \neq 1/2$, the variance depends on $\psi(x_i)$, complicating the power and sample size calculations.

Parallel-group analysis with unequal allocation ratios

Similarly, for the parallel-group analysis, let the allocation ratio to the treatments be $q : (1 - q)$, where $q = \Pr(A_{i1} = 1)$. We define the transformed treatment variable A'_{i1}:

$$A'_{i1} = \frac{A_{i1}}{2qA_{i1} + (1 - A_{i1})}. \tag{S.3}$$

Defining the transformed response as $Z'_i = 2Y_{i1}A'_{i1}$, the expectation of this transformed variable is:

$$\mathrm{E}[Z'_i] = \mathrm{E}[A'_{i1}A_{i1}]\theta(x_i) + 2\mathrm{E}[A'_{i1}]\mathrm{E}[m_i + e_{i1}] = \theta(x_i), \tag{S.4}$$

where the treatment effect for individual i is denoted by $\theta(x_i) = \theta_0 + \theta_1 x_i$. This confirms that Z'_i is an unbiased estimator of $\theta(x_i)$ even under unequal allocation, since $\mathrm{E}[A'_{i1}] = 0$ and $\mathrm{E}[A'_{i1}A_{i1}] = 1$.

While the transformation Z'_i provides an unbiased estimator of $\theta(x_i)$, the variance of Z'_i under unequal allocation ($q \neq 1/2$) is more complex and depends on the treatment effect $\theta(x_i)$.

Using the properties of A'_{i1} derived in the proof of $\mathrm{E}[Z'_i]$ and the independence between A_{i1} and (m_i, e_{i1}), the variance can be derived as:

$$\mathrm{Var}(Z'_i) = \left\{\frac{1}{4q(1-q)} - 1\right\}\theta(x_i)^2 - \frac{(1-2q)\mu_m}{2q(1-q)}\theta(x_i) + \frac{\sigma_m^2 + \mu_m^2 + \sigma_e^2}{q(1-q)} \tag{S.5}$$

When $q = 1/2$, this expression simplifies to $\mathrm{Var}(Z'_i) = 4(\sigma_m^2 + \mu_m^2 + \sigma_e^2)$, which is constant and independent of the treatment effect $\theta(x_i)$. However, for $q \neq 1/2$, the variance depends on $\theta(x_i)$ and μ_m, complicating the power and sample size calculations.

S3.6 Correspondence with conventional crossover design notation

To clarify the relationship between our proposed model and the standard notation used in crossover designs, and to justify the introduction of random effects, we provide a mapping

based on the conventional cell means model (Jones and Kenward, 2014).

Conventional 2×2 crossover model

Let i' denote the sequence ($i' = 1$: A $\to$ B; $i' = 2$: B $\to$ A) and j denote the period ($j = 1, 2$). Let $k' = 1, \ldots, n_{i'}$ index the subjects within sequence i'. Let $d_{i'j} \in \{\text{A}, \text{B}\}$ be the treatment administered in sequence i' at period j. The conventional model is written as:

$$Y_{i'jk'} = \mu + \pi_j + \tau_{d_{i'j}} + \lambda_{d_{i'(j-1)}} + s_{i'k'} + e_{i'jk'}, \tag{S.6}$$

where μ is the overall mean, π_j is the period effect, τ represents the direct treatment effect, λ represents the carryover effect with $\lambda_{d_{i'0}} = 0$, $s_{i'k'}$ is the subject effect, and $e_{i'jk'}$ is the error term.

One-parameter representation

To ensure parameter identifiability, the following zero-sum constraints are typically imposed:

$$\pi_1 + \pi_2 = 0, \quad \tau_A + \tau_B = 0, \quad \lambda_A + \lambda_B = 0. \tag{S.7}$$

Under the constraints in (S.7), there exist scalars π, τ, λ such that:

$$\pi_1 = \pi,\ \pi_2 = -\pi; \quad \tau_A = \tau,\ \tau_B = -\tau; \quad \lambda_A = \lambda,\ \lambda_B = -\lambda. \tag{S.8}$$

We introduce the following indicator variables:

- Period variable: $P_{i'jk'} = 1$ if $j = 1$, and -1 if $j = 2$.
- Treatment variable: $T_{i'jk'} = 1$ if $d_{i'j} = \text{A}$, and -1 if $d_{i'j} = \text{B}$.
- Carryover variable: $C_{i'jk'} = 0$ if $j = 1$, and $T_{i'(j-1)k'}$ if $j = 2$.

Since k' indexes the individual within a sequence, once the sequene index i' and period j are fixed, the model can be rewritten for the i-th individual in the whole population as:

$$Y_{ij} = \mu + \pi P_{ij} + \tau T_{ij} + \lambda C_{ij} + s_i + e_{ij}. \tag{S.9}$$

General form of heterogeneous treatment effects

We consider separating individual information into the marker of interest (effect modifier), x_i, and other background factors (observed or unobserved), w_i. We model the heterogeneity of treatment and carryover effects explicitly as functions of x_i, while the effects of other factors w_i are absorbed into individual-specific effects. The general model considering these factors is:

$$Y_{ij} = \mu(z_i) + \pi(z_i)P_{ij} + \tau(x_i)T_{ij} + \lambda(x_i)C_{ij} + u_i(z_i) + e_{ij}, \tag{S.10}$$

where $\mu(w_i)$ and $\pi(w_i)$ represent the baseline and period effects dependent on w_i, $\tau(x_i)$ and $\lambda(x_i)$ represent the treatment and carryover effects dependent on x_i, and $u_i(w_i)$ is the random subject effect.

Specification for binary markers and correspondence to proposed model

When the marker x_i is binary (0/1), the treatment and carryover effects can be expressed as $\tau(x_i) = \tau_0 + \tau_1 x_i$ and $\lambda(x_i) = \lambda_0 + \lambda_1 x_i$. Substituting these into (S.10) yields:

$$Y_{ij} = \mu(z_i) + \pi(z_i)P_{ij} + (\tau_0 + \tau_1 x_i)T_{ij} + (\lambda_0 + \lambda_1 x_i)C_{ij} + u_i(z_i) + e_{ij}. \tag{S.11}$$

Our proposed model is:

$$Y_{ij} = m_i + \frac{A_{ij}}{2}(\theta_0 + \theta_1 x_i) + \phi_{ij}\left\{t_i + \frac{A_{ij}}{2}(\gamma_0 + \gamma_1 x_i)\right\} + e_{ij}. \tag{S.12}$$

Comparing the variables, $T_{ij} = A_{ij}$ and $C_{ij} = -\phi_{ij}A_{ij}$ (since carryover exists only in

period 2). This establishes the following relationship between the parameters:

$$\tau_0 = \frac{\theta_0}{2}, \quad \tau_1 = \frac{\theta_1}{2}, \quad \lambda_0 = -\frac{\gamma_0}{2}, \quad \lambda_1 = -\frac{\gamma_1}{2}. \tag{S.13}$$

Furthermore, using the relationship $P_{ij} = 1 - 2\phi_{ij}$, the terms dependent on w_i in (S.11) correspond to our random effects as follows:

$$m_i = \mu(w_i) + \pi(w_i) + u_i(w_i), \tag{S.14}$$

$$t_i = -2\pi(w_i). \tag{S.15}$$

Thus, our proposed model effectively captures the x_i-independent variability (due to w_i) through the random effects (m_i, t_i), while explicitly modeling the heterogeneous treatment effects due to x_i via fixed parameters.

References

Jones, B. and Kenward, M. G. (2014). *Design and Analysis of Cross-Over Trials*. Chapman and Hall/CRC.
